# A Survey of Essential Genome Stability Genes Reveals That Replication Stress Mitigation Is Critical for Peri-Implantation Embryogenesis

**DOI:** 10.3389/fcell.2020.00416

**Published:** 2020-05-29

**Authors:** Georgia R. Kafer, Anthony J. Cesare

**Affiliations:** Genome Integrity Unit, Children’s Medical Research Institute, The University of Sydney, Westmead, NSW, Australia

**Keywords:** early development, embryology, pluripotency, DNA damage response, DNA repair, DNA replication, replication stress response

## Abstract

Murine development demands that pluripotent epiblast stem cells in the peri-implantation embryo increase from approximately 120 to 14,000 cells between embryonic days (E) 4.5 and E7.5. This is possible because epiblast stem cells can complete cell cycles in under 3 h *in vivo*. To ensure conceptus fitness, epiblast cells must undertake this proliferative feat while maintaining genome integrity. How epiblast cells maintain genome health under such an immense proliferation demand remains unclear. To illuminate the contribution of genome stability pathways to early mammalian development we systematically reviewed knockout mouse data from 347 DDR and repair associated genes. Cumulatively, the data indicate that while many DNA repair functions are dispensable in embryogenesis, genes encoding replication stress response and homology directed repair factors are essential specifically during the peri-implantation stage of early development. We discuss the significance of these findings in the context of the unique proliferative demands placed on pluripotent epiblast stem cells.

## Introduction

### Overview

Pluripotent cells in early mammalian embryos proliferate at a phenomenal rate. This is necessary to maintain embryo growth and reach critical developmental milestones within defined temporal windows. Because all somatic tissues are derived from these early pluripotent precursors, it is critical that genome integrity is maintained during early development. Embryonic pluripotent stem cells are thus subjected to unique challenges to maintain their DNA health. To elucidate which genome stability pathways are essential for early development we probed the Mouse Genome Informatics Gene Ontology Project (MGI-GO) database (Bult et al., 2019). Within MGI-GO we identified 347 genes grouped within the ontologies of major DNA repair pathways (MGI-GO designations: DNA damage checkpoint, nucleotide excision repair, mismatch repair, base excision repair, homologous recombination, and non-homologous end joining). Of these genes, we identified 297 with a validated mouse knockout. From these 297 murine models, only 108 gene knockouts were lethal during embryonic development (Supplementary Table S1). Within the grouping of 108 embryonic lethal genes, 10 knockouts were lethal during preimplantation development prior to E4.5 (Table 1), and 36 knockouts were lethal during somite stages from E8.5 (Supplementary Table S1). Notably, most of the targeted genes that conferred embryonic lethality, 62 genes, did so specifically during the period of rapid cell proliferation occurring with peri-implantation development (E4.5 to E8.5) (Table 2). Below we briefly review pre- and peri-implantation murine development before considering the function of essential genome stability factors across the early stages of embryonic development. Finally, we discuss why the unique cells of the peri-implantation embryo appear to specifically require replication stress response factors for cell viability.

**TABLE 1:** Genome stability factors essential for preimplantation development.

**Targeted gene**	**Lethal at**	**Phenotype details**	**Gene Function**	**References**
*Ercc2 (Xpd)*	E1.5	Mutant embryos do not implant. *In vitro* culture indicates embryo failure at the 2-cell stage.	NER*	de Boer et al., 1998
*Dtl*	E2.5	Embryos develop to the morula stage but do not form blastocysts.	CC, DDR	Liu et al., 2007
*Pcna*	E2.5	Embryos do not survive to the blastocyst stage.	Rep, BER, NER, MMR	Roa et al., 2008
*Rpa1*	E2.5	Morula form but do not progress to blastocysts.	Rep, HDR, NER, BER, MMR	Wang et al., 2005
*Wee1*	E2.5	Morula form but do not progress to blastocysts.	CC, DDR	Tominaga et al., 2006
*Nop53 (Pict1)*	Before E3.5	Mutant embryos develop morphologically typical morula, but do not form well-structured blastocysts.	CC, DDR*	Sasaki et al., 2011
*Cdk1*	Before E3.5	Morula form but do not progress to blastocysts.	CC, DDR	Diril et al., 2012
*Plk1*	E3.5	Mutant embryos develop to the morula stage but do not form viable blastocysts *in vitro*.	CC, DDR	Lu et al., 2008
*Xab2*	E3.5	Mutant embryos form morula and show some signs of compaction, but do not form blastocysts.	NER*	Yonemasu et al., 2005
*Pot1a*	After E3.5	Morula are recovered and can be cultured but do not form blastocysts.	Telomere	Hockemeyer et al., 2006

Key: (E) = Embryonic day; DDR = DNA damage response/damage sensing; CC = cell cycle control; Rep = DNA replication and/or replication stress; HDR = Homologous directed repair; EJ = End joining pathways; BER = Base excision repair; NER = Nucleotide excision repair; MMR = Mismatch repair; Telomere = telomere protein. Gene functions are cross-referenced with the literature and may diverge from the MGI-GO categorization. Factors labeled with * indicate when the gene encodes a protein with additional functions outside genome stability that likely contribute to embryonic lethality.

**TABLE 2:** Genome stability factors essential for peri-implantation development.

**Targeted gene**	**Lethal at**	**Phenotype details**	**Gene function**	**References**
*Zfp830 (Omcg1)*	E3.5	Knockouts blastocysts form but fail to outgrow and do not induce an implantation response *in vivo*.	CC, Rep, DDR*	Artus et al., 2005
*Actr2*	After E3.5	Lethal after blastocyst stage.	HDR*	Zhang et al., 2007
*Cdc7*	After E3.5	Blastocysts form and can hatch. Outgrowth assays demonstrate with *in vitro* culture knockout embryos have a small ICM population by E5.5.	CC, Rep, DDR	Kim et al., 2002
*Chek1*	After E3.5	Blastocysts form *in vivo* but are resorbed following implantation. *In vitro* cultured blastocysts have elevated apoptosis in ICM populations.	Rep, DDR	Takai et al., 2000 Zaugg et al., 2007
*Atr*	E4.5	Blastocysts form, hatch, and show evidence of initiating implantation *in utero*. ICM outgrowth assays are consistent with wildtype embryos, but ICM populations succumb to apoptosis with continued culture.	CC, Rep, DDR, HDR	de Klein et al., 2000 Brown and Baltimore, 2000 Murga et al., 2009
*Cdc25A*	E4.5	Blastocysts form but suffer from impaired hatching ability.	CC, Rep	Ray et al., 2007
*Ctip (Rbbp8)*	E4.5	Embryos form blastocysts but fail to form an egg cylinder. Cells appear to suffer from reduced DNA synthesis.	Rep, HDR	Chen et al., 2005
*Recql4*	From E4.5	Outcomes are dependent on the genetic manipulation. Deletion of exons 5–8 are lethal during gastrulation, deletion of exon 13 results in lethality just after birth.	Rep, HDR	Hoki et al., 2003
*Rint1*	From E4.5	Null embryos implant *in vivo* but are severely developmentally delayed and resorbed from E6.5. The ICM and trophoblasts initially outgrow *in vitro*, but proliferation fails after 6 days.	HDR*	Lin et al., 2013
*Thoc1*	From E4.5	Embryos form a blastocyst, hatch, and attach in *in vitro*, but suffer from reduced proliferation of the ICM after several days in culture. No embryos are recovered *in vivo* before E8.5, but decidua are present, suggesting the embryos die during gastrulation.	CC, HDR, DDR*	Wang et al., 2006
*Apex1*	After E4.5	Blastocysts form and attach but die soon after.	Rep, BER	Xanthoudakis et al., 1996
*Cdc45*	After E4.5	Blastocysts form and can hatch. Blastocyst outgrowth assays demonstrate that after several days of culture the ICM mass is smaller or not present.	CC, Rep	Yoshida et al., 2001
*Cdk7 (Cdks)*	After E4.5	Decidual resorption at peri-implantation stages *in vivo. In vitro* cultured embryos demonstrate increased apoptosis in the blastocyst and severely reduced ICM proliferation.	CC, Rep	Ganuza et al., 2012
*Fen1*	After E4.5	Blastocysts form and hatch but are compromised during peri-implantation.	Rep, EJ, BER,	Larsen et al., 2003
*Gins4 (Sld5)*	After E4.5	Embryos form blastocysts that hatch and implant. Development is compromised after implantation as embryos do not form egg cylinders. Embryos attach in outgrowth assays, but after 2 days of culture the ICM becomes compromised.	Rep	Mohri et al., 2013
*Hinfp*	After E4.5	Knockout blastocysts can form, hatch, and attach, but embryos do not survive past E6.5.	CC, Rep*	Xie et al., 2009
*Mdm2*	After E4.5	Embryos can implant but are quickly resorbed.	DDR	Jones et al., 1995
*Mnat1*	After E4.5	Blastocysts form that are indistinguishable from wildtype littermates, but no embryos are found after gastrulation.	CC, Rep*	Rossi et al., 2001
*Pold3*	After E4.5	Blastocysts form, hatch, and attach *in vitro* but show reduced outgrowth compared to wildtype embryos. *In vivo*, no embryos are recovered at E7.5 consistent with embryo failure during gastrulation.	Rep, NER, TLS	Zhou et al., 2018
*Prpf19*	After E4.5	Same as above.	CC, Rep, DDR, HDR	Fortschegger et al., 2007
*Ptpn11*	After E4.5	Same as above.	CC*	Yang et al., 2006
*Topbp1*	After E4.5	Blastocysts form and hatch but are unable to attach to the tissue culture dish *in vitro*.	CC, Rep, DDR, HDR	Jeon et al., 2011
*Yy1*	From E5.0	Knockout blastocysts, form, hatch, and outgrow normally *in vitro.* However, *in vivo*, embryos fail to form and egg cylinder and die during gastrulation.	DDR, HDR*	Affar El et al., 2006
*Rad51*	Before E5.5	Blastocysts appear normal, but post-implantation embryos fail to properly develop an amniotic cavity, display no discernible mesoderm, have a reduced proliferation rate, and elevated apoptosis.	Rep, HDR	Lim and Hasty, 1996 Tsuzuki et al., 1996
*Thoc5*	Before E5.5	Embryos are not recovered from the uterus from E5.5 suggestive of death during preimplantation or gastrulation.	CC, HDR, DDR*	Mancini et al., 2010
*Timeless*	From E5.5	Embryos display severe cellular disorganization during gastrulation.	Rep	Gotter et al., 2000
*Brca1*	E5.5	Growth arrest and impaired proliferation resulting in resorption.	Rep, HDR	Hakem et al., 1996 Ludwig et al., 1997
*Mre11A*	E5.5	Embryos compromised during gastrulation.	Rep, HDR, DDR, EJ	Xiao and Weaver, 1997 Theunissen et al., 2003 Buis et al., 2008
*Nbn (Nbs1)*	E5.5	Blastocysts form and hatch, but embryos are smaller after implantation and are resorbed around gastrulation.	Rep, DDR, HDR, EJ	Zhu et al., 2001
*Ube2N*	E5.5	Embryos are never recovered, and timed mating suggests that embryos die during gastrulation.	HDR	Fukushima et al., 2007
*Rad50*	Before E6.0	Embryos are abnormal from gastrulation onset and are resorbed.	Rep, DDR, HDR, EJ	Luo et al., 1999
*Fh1*	E6.0	Embryos do not appear to grow past the early egg cylinder stage.	NHEJ*	Pollard et al., 2007
*Blm*	E6.5	Reduced embryo size, epiblast population and mesoderm population. The primitive streak forms but there is increased apoptosis throughout the embryo.	HDR, Rep	Chester et al., 1998
*Cops5*	E6.5	Gastrulating embryos are smaller and cannot develop all germ layers. Embryos display elevated apoptosis.	CC, DDR*	Tomoda et al., 2004
*Kdm1A*	E6.5	Blastocysts form, hatch, and attach *in vitro*, but are developmentally stunted from E6.5.	DDR, HDR*	Foster et al., 2010
*Usp7*	E6.5	Embryos die during gastrulation due to reduced proliferation.	Rep, NER	Kon et al., 2010
*Xrcc1*	E6.5	Epiblast cell numbers are reduced in early gastrulating embryos due to apoptosis and slow proliferation, which impacts lineage specification.	EJ, BER	Tebbs et al., 1999
*Pold1*	Before E7.5	Knockout embryos form blastocysts at E4.5 but no embryos are retrieved at E7.5.	Rep, BER, NER, MMR	Uchimura et al., 2009
*Cul4A*	Before E7.5	Embryos form blastocysts *in vitro* that hatch from the zona pellucida with no ICM or trophoblast compromise. No characterization of lethality presented.	CC, Rep, DDR, NER	Li et al., 2002
*Dna2*	Before E7.5	No embryos retrieved after E7.5. The cause of lethality was not investigated further.	Rep, HDR, BER	Lin et al., 2013
*Rif1*	From E7.5	Null embryos are lethal from gastrulation in some genetic backgrounds but are viable in different mouse strains. Dimorphism in embryo survivability observed where male knockouts survive but female knockouts do not.	Rep, HDR	Chapman et al., 2013
*Bard1*	E7.5	Embryos do not develop past egg cylinder stage.	Rep, HDR	McCarthy et al., 2003 Shakya et al., 2008
*Brca2*	E7.5	Reduced embryo size and persisting Oct4 positive egg cylinder indicates retarded dermal commitment of epiblast cells.	Rep, HDR	Sharan et al., 1997
*Hus1*	E7.5	Gastrulating embryos appear normal but are smaller than wildtype littermates. Development becomes severely delayed from mid-gastrulation.	Rep, DDR, BER, NER	Weiss et al., 2000
*Ino80*	E7.5	Knockout embryos implant as deciduas are found at E7.5, but embryos are resorbed during organogenesis.	Rep, DDR, HDR*	Min et al., 2013
*Lig3*	E7.5	Embryos are smaller from gastrulation onset and do not develop past mid-gastrulation.	Rep, EJ, BER, NER	Puebla-Osorio et al., 2006
*Ptip*	E7.5	Gastrulating embryos display increased apoptosis. Embryos can be recovered in the early stages of organogenesis but are poorly formed.	Rep, DDR, EJ, HDR	Cho et al., 2003
*Rad51B (Rec2)*	E7.5	Knockout embryos implant, but do not develop pro-amniotic cavities and are resorbed by E7.5.	HDR	Shu et al., 1999
*Uvrag*	E7.5	Knockout mice die by E7.5 but no additional data is provided as to the cause of the lethality.	EJ*	Afzal et al., 2015
*Wdr48 (Uaf1)*	E7.5	Knockout mice die during gastrulation.	Rep, HDR	Park et al., 2013
*Ercc3 (Xpb)*	Before E8.5	No healthy embryos are recovered by early organogenesis. Embryos may be compromised earlier but no additional details are provided.	NER*	Andressoo et al., 2009
*Ddb1*	Before E8.5	Same as above.	CC, Rep, DDR, NER	Cang et al., 2006
*Palb2*	E8.5	Embryos are developmentally retarded after gastrulation and die during organogenesis.	Rep, HDR	Bowman-Colin et al., 2013
*Rad51C*	E8.5	Knockout embryos implant but are developmentally delayed, resulting in impaired gastrulation and resorption from E8.5.	Rep, HDR	Kuznetsov et al., 2009
*Rev3L*	After E8.5	Blastocysts form but do not thrive *in vitro*. Embryos are smaller than wildtype littermates during gastrulation.	HDR, TLS	Bemark et al., 2000
*Pnkp*	After E9.0	Embryos are not recovered from the onset of organogenesis. No further details are provided as to the cause of lethality.	EJ, BER	Shimada et al., 2015
*Nipbl (Scc2)*	Before E9.5	No knockout mice are recovered from the onset of organogenesis, but no additional data is provided.	Rep, DDR, HDR, EJ	Smith et al., 2014
*Srpq*	Before E9.5	Knockouts cannot be recovered from the start of organogenesis, implying that embryos die during gastrulation.	HDR*	Takeuchi et al., 2018
*Ppp4C*	Before E9.5	Same as above.	HDR	Shui et al., 2007
*Rnaseh2B*	From E9.5	Knockouts are smaller than littermates at gastrulation conclusion and die early in organogenesis.	Rep, DDR	Hiller et al., 2012
*Syf2*	E9.5	Knockouts do not survive organogenesis. Embryos can implant but gastrulation does not proceed normally.	CC, Rep	Chen et al., 2012
*Nsmce2*	Before E10.5	Knockout mice are not recovered from the uterus by E10.5. Embryos recovered at E2.5 appear normal but do not thrive *in vitro*. The cause of embryonic failure is not reported.	HDR	Jacome et al., 2015

Key: (E) = Embryonic day; DDR = DNA damage response/damage sensing; CC = cell cycle control; Rep = DNA replication and/or replication stress; HDR = Homologous directed repair; EJ = End joining pathways; BER = Base excision repair; NER = Nucleotide excision repair; MMR = Mismatch repair; Telomere = telomere protein; TLS = Translesion synthesis. Gene functions are cross-referenced with the literature and may diverge from the MGI-GO categorization. Factors labeled with * indicate when the gene encodes a protein with additional functions outside genome stability that likely contribute to embryonic lethality.

### Early Murine Development

**FIGURE 1:**
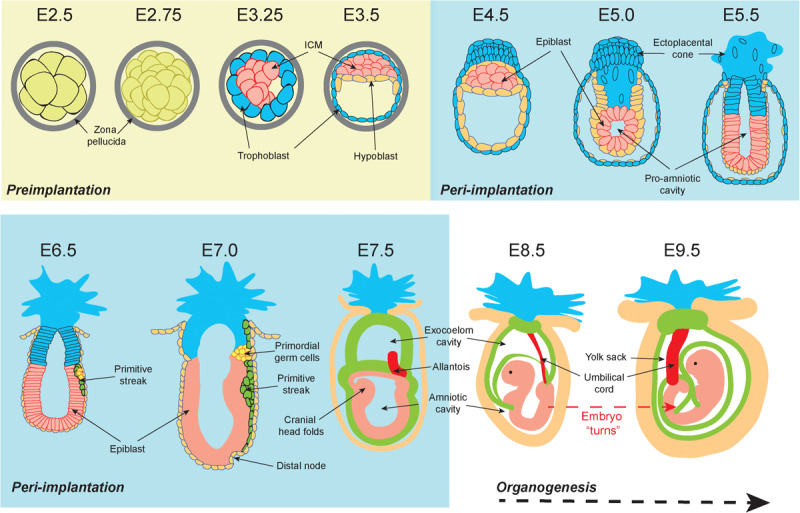
Graphical representation of the early stages of murine development. At embryonic day 2.5 (E2.5) totipotent cells within the preimplantation morula are encased within a protective zona pellucida shell. From E3.25 the blastocoel cavity opens, and the embryonic cells differentiate into the inner cell mass (ICM) (pink) and the outer trophoblast (blue). By E3.5 the hypoblast (orange) has developed. During peri-implantation the zona pellucida shell is lost by E4.5 and the ICM are now termed “epiblast cells” (pink). The outer trophoblast cell layer continues to encapsulate the embryo and expands to form the ecto-placental cone that is the precursor for placental tissues. From E5.0 a pro-amniotic cavity opens and elongates. By E6.5 the embryo has implanted, the primitive streak (green) emerges, and the primordial germ cell populations form (yellow). The primitive streak continues to elongate and is accompanied by the formation of the distal node at E7.0. At E7.5 the primitive streak derived mesoderm extends around the embryo and divides the pro-amniotic cavity into the exocoelom cavity and the amniotic cavity. At E8.5 the exocoelom becomes the yolk sack. Between E8.5 and E9.5 the embryo turns, resulting in complete envelopment of the embryo within the supporting extraembryonic tissues. Embryo depictions are not to scale.

Embryonic development consists of a series of events occurring in chronological progression. Murine development takes 19 to 20 days depending on mouse strain (Figure 1; Murray et al., 2010). Preimplantation development occurs between fertilization (E0) and the initiation of embryo implantation in the uterine wall (around E4.0). Following fertilization, embryonic cells are uniformly totipotent and identical until formation of the morula at E2.5 (Condic, 2014) (see Box 1 for a detailed explanation of cell potency). Within the morula cells undergo polarization and compaction (Humięcka et al., 2017). By E3.0 the embryo forms a blastocyst structure containing two distinct cell populations; an outer layer of multipotent trophectoderm cells that eventually derive the placental tissues, and an inner group of pluripotent inner cell mass (ICM) cells which primarily serve as precursors for the embryo proper (Morris et al., 2010).

**FIGURE 2:**
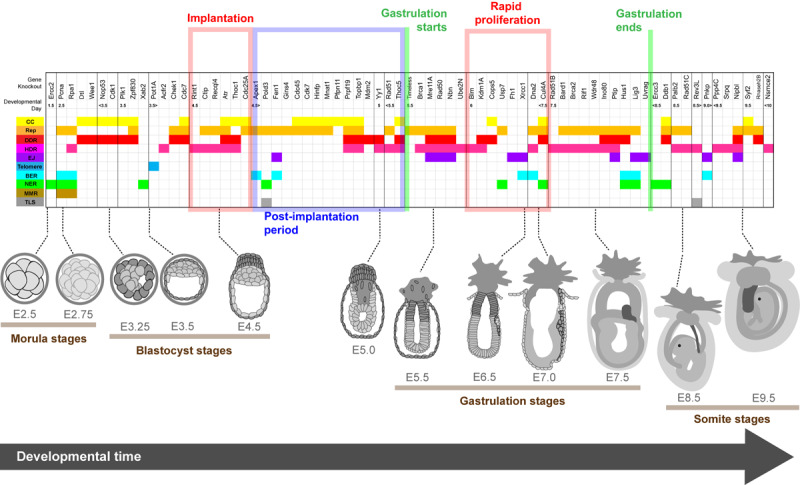
Lethality onset in embryos deleted for genome stability factors relative to developmental stage. Spatiotemporal heat map illustrating the timing of lethality for embryos lacking genome stability factors essential for early development. Genes are listed above and organized temporally relative to the reported timing of embryo compromise. Graphical depiction of normal embryo structure at the time of lethality is shown below and key developmental periods are highlighted. Gene functions according to the literature are color coded as follows: cell cycle regulation (CC, yellow); DNA replication and/or the replication stress response (Rep, orange); DNA damage response (DDR, red); Homology directed repair (HDR, pink); canonical and alternative non-homologous end joining (EJ, purple); telomere biology (Telomere, blue); base excision repair (BER, cyan); nucleotide excision repair (NER, green); mismatch repair (MMR, brown) and translesion synthesis (TLS, gray). Embryo depictions are not to scale.

**Box 1.**

Potency states. Totipotent cells are present in the embryo between fertilization and morula formation. They can give rise to any cell type from any stage of the animal’s life, including germ and placental cells. Pluripotent cells exist in the inner cell mass (ICM) and epiblast region of the developing embryo from the blastocyst stage to immediately prior to organogenesis. Pluripotent cells can give rise to any cell of dermal lineage (mesoderm, endoderm or ectoderm) but not placental or germ cells. Multipotent cells exist in newly formed tissues or organs and can develop into a limited number of cell types within their original dermal lineage.

Peri-implantation is the developmental period from implantation to organogenesis (E4.0 to E8.5). Implantation begins at E4.0 when the free-floating embryo contains 64 cells (Behringer et al., 2014). At this point, the embryo loses its glycoprotein zona pellucida shell and the outer trophoblast attaches to the uterine wall. Single cell transcriptomics demonstrate that at E4.0 the ICM has differentiated into two pluripotent cell types: the primitive endoderm (PrE, sometimes referred to as the hypoblast) and the epiblast (Mohammed et al., 2017). Peri-implantation development is associated with exceptionally rapid cell proliferation during “gastrulation,” which begins at E5.5 as the embryo elongates and a luminal pro-amniotic cavity opens in the center of the epiblast cell mass (Snow, 1976). At E6.5 the primitive streak emerges from the epiblast, and from the epiblast all three dermal lineages of the conceptus will arise (Tam et al., 1993). These are the ectoderm (nervous system and skin precursor), endoderm (gut precursor), and mesoderm (precursor for all other tissues) (Watson and Tam, 2001). Because continued gastrulation requires a critical mass of 1000 cells, delays in epiblast proliferation at E6.5 may result in the embryo forming a small primitive streak but developing no further (Snow and Tam, 1979; Tam, 1989; Power and Tam, 1993).

Between E6.5 and E7.5 the rate of epiblast proliferation is exceptionally pronounced, with some cell cycles completed in 2.2 h (Snow, 1977). Elongation of the primitive streak between E6.5 and E7.0 is associated with the first evidence of epithelial to mesenchymal transition (EMT) (Carver et al., 2001). EMT mobilizes epiblast cells to form the endoderm and mesoderm lineages, while cells that do not undergo EMT become the ectoderm (Acloque et al., 2009). At E7.5 the mouse embryo contains over 14,000 cells derived from epiblast progenitors. Proliferation slows at this point resulting in an increased average cell cycle duration of 8.1 h (Snow, 1977). There is evidence to suggest a that a specialized “proliferative zone” with sub 4-h cell cycles is maintained within the primitive streak after E7.5, however, definitive evidence remains to be substantiated (Tam et al., 2013).

### Genome Instability Differentially Impacts Cell Viability in Pre- and Peri-Implantation Embryos

Embryonic lethality implies that one or more cell populations are compromised during development at the time of embryo failure. This may arise through DNA damage or genome instability, with multiple lines of evidence indicating that preimplantation embryos are more resistant to genome perturbations than peri-implantation embryos. For example, gamma irradiation-induced DNA damage at E6.5 or E7.5 confers apoptosis specifically within the epiblast (Heyer et al., 2000). Conversely, apoptosis levels are low in preimplantation embryos irradiated between E3.5 and E5.5, or during organogenesis from E8.5. It is therefore the epiblast that is particularly sensitive to these types of DNA lesions. Similarly, despite tetraploidy driving additional chromosomal instability in preimplantation cells (Paim and FitzHarris, 2019) chimeric mouse embryos with a mixture of diploid and tetraploid cells will develop through preimplantation before dying during peri-implantation (Horii et al., 2015). Notably, tetraploid sensitivity *in vivo* appears to be specific to the epiblast as embryos with tetraploid trophoblast cells and diploid epiblast cells can generate live pups (Wen et al., 2017). Mouse embryos containing a mixture of diploid and aneuploid cells will also develop to peri-implantation before the aneuploid cells are specifically depleted in the epiblast through apoptosis (Bolton et al., 2016).

As with somatic tissues, the tumor suppressor *TP53* (p53) plays a central role regulating stem cell outcomes following genomic insult. p53 orchestrates growth arrest or apoptosis following activation of the DNA damage response (Mello and Attardi, 2018). Concordantly, inhibiting p53-dependant signaling pathways enables chimeric embryos made from tetraploid preimplantation murine embryonic stem cells (mESCs) to survive until birth (Horii et al., 2015). Deleting *TP53* also reduced apoptosis levels in irradiated E6.5 embryos (Heyer et al., 2000) and extended the survival of embryos co-deleted for essential DNA repair factors (Jones et al., 1995; Haupt et al., 1997; Ludwig et al., 1997; Kim et al., 2002; McCarthy et al., 2003; Cang et al., 2006; Reinhardt and Schumacher, 2012). Not surprisingly, *TP53* was identified as a critical mediator of apoptosis in the gastrulating epiblast (Laurent and Blasi, 2015). However, when activated in pluripotent stem cells, p53 also influences the expression of pluripotency factors to regulate differentiation (Lin et al., 2005; Li et al., 2012; Akdemir et al., 2014; Jain et al., 2016). p53 therefore functions through canonical and unique pathways in early development to regulate cellular outcomes. This highlights that our classic understanding of genome stability pathways may not strictly apply to early development or certain pluripotent cell types (Zaveri and Dhawan, 2018).

## DNA Damage Response and Repair Pathways

### Replication Stress Response

Somatic mammalian cells prepare for DNA replication in G1 phase by licensing replication origins and loading inactive Cdc45-MCM-GINS replicative helicase complexes (Bleichert, 2019; Miller et al., 2019). Cyclin dependent kinase activity promotes E2F transactivation to initiate replication at the G1/S transition (Kent and Leone, 2019). Replication then proceeds throughout the S-phase with origins firing in temporal coordination and DNA synthesis occurring across the entirety of the genome (Burgers and Kunkel, 2017; Limas and Cook, 2019). Intrinsic and extrinsic factors may disrupt replication fork processivity: a phenomenon known as “replication stress” (Zeman and Cimprich, 2014). Replication stress is sensed through the accumulation of RPA binding to its single strand DNA (ssDNA) substrate (Bhat and Cortez, 2018). When replication stress stalls DNA synthesis the replicative helicase continues to unwind its substrate exposing ssDNA for RPA coating (Byun et al., 2005). ATR kinase is the master regulator of the replication stress response (Saldivar et al., 2017). RPA coated ssDNA recruits ATR and its associated protein ATRIP (Cortez et al., 2001) to stalled replication forks through parallel pathways mediated by TopBP1 and ETAA1 (Kumagai et al., 2006; Bass et al., 2016; Haahr et al., 2016). Once localized to the stalled fork, ATR is activated and propagates a signaling cascade resulting in engagement of the replication stress response. This includes activation of the downstream effector CHK1 kinase to arrest S phase until replication stress is resolved (Zhang and Hunter, 2014). During the replication stress response, stalled replication forks are often remodeled into a four-way structure and protected before engaging one of many diverse repair mechanisms dependent upon the underlying stress the fork encountered (Quinet et al., 2017; Cortez, 2019).

If replicative stress is unresolved, arrested replication forks may collapse into one-ended double strand breaks (DSBs) (Ait Saada et al., 2018). Additionally, persistent replication stress can result in under-replicated DNA persisting through S-phase, the second growth (G2) phase, and into the mitotic (M) phase of the cell cycle (Mankouri et al., 2013). Specialized repair mechanisms address replication defects carried into mitosis (Minocherhomji et al., 2015), during which time the canonical DSB repair pathways are inhibited (Orthwein et al., 2014). Replication defects passed into mitosis can confer chromosome segregation errors resulting in aneuploidy (Burrell et al., 2013; Wilhelm et al., 2019), or if severe mitotic death (Masamsetti et al., 2019). If a replication stressed cell escapes mitosis this is often evident in the daughter cells where the under-replicated DNA is present as a scar in the first growth phase (G1) that is repaired in the subsequent S phase (Lukas et al., 2011; Spies et al., 2019).

### Double Strand Break Repair

DSBs are a major threat to genome stability because a failure in repair may result in loss of an entire chromosome arm (Scully et al., 2019). Additionally, chromosome segregation errors resulting from inadequate DSB repair are rife with implications for genome instability, including chromothripsis and kataegis (Maciejowski et al., 2015; Ly and Cleveland, 2017). DSBs are sensed by Ku70/80 (Gottlieb and Jackson, 1993; Ochi et al., 2015), PARP1 (Ali et al., 2012; Liu C. et al., 2017), and the MRN (MRE11, RAD50 and NBS1) complex (Lee and Paull, 2005; Stracker and Petrini, 2011). MRN facilitates recruitment of the ATM kinase to DSBs (Uziel et al., 2003) while Ku70/80 recruits a related kinase DNA-PKcs (Hnizda and Blundell, 2019). Once recruited to the break, ATM is activated and engages downstream DDR pathways (Dupre et al., 2006; Maréchal and Zou, 2013). This includes phosphorylation of the histone variant H2AX (termed γ-H2AX when phosphorylated) within the break adjacent chromatin and assembly of factors at the break locus to facilitate repair (Rogakou et al., 1998). ATM activation also results in growth arrest, including activation of the p53 pathway (Banin et al., 1998). Subsequent repair of DNA breaks is then orchestrated by homology directed repair (HDR) or end-joining (EJ) pathways.

Homology directed repair utilizes the homologous sister chromatid as a template for repair and is thus limited to the S and G2 cell cycle phases (Hustedt and Durocher, 2016). HDR initiates with resection at the broken DNA end to provide a 3′ ssDNA overhang for insertion into homologous regions of the sister chromatid (Symington, 2016). Once resection occurs at a canonical two-ended DSB, a series of enzymatic steps facilitate strand-invasion of the broken end into the sister chromatid, formation of a displacement-loop, template copying from the invaded DNA end, potential formation of a “Holliday Junction,” and resolution of strand invasion (Scully et al., 2019). HDR factors also function within the replication stress response though diverse mechanisms including the remodeling and stabilization of stressed replication forks into four-way structures prior to repair (Neelsen and Lopes, 2015). Additionally, HDR factors participate in resolving one-ended DSBs that arise from collapsed replication forks to restart replication (Ait Saada et al., 2018). HDR is thus intrinsically linked to the replication stress response.

Conversely, EJ involves the covalent ligation of broken DNA ends to repair DSBs and thus does not require an additional chromatid template. End-joining mechanisms function in S and G2, but notably are the only DSB repair pathway available in G1 (Chang et al., 2017). Classical non-homologous end joining (c-NHEJ) directly ligates DNA ends, and if unregulated can also drive chromosome translocations (Ghezraoui et al., 2014) or telomere fusions (Celli et al., 2006; Van Ly et al., 2018). Cells can also engage alternative non-homologous end joining (alt-NHEJ), where DNA resection creates 3′ overhangs to align the DNA ends for repair through microhomology (Sfeir and Symington, 2015). This often requires a fill-in reaction by the error prone polymerase Pol θ which can introduce errors into the repaired sequence (Mateos-Gomez et al., 2015; Mateos-Gomez et al., 2017).

### Nucleotide and Base Damage

In addition to DNA breaks, cells must also contend with damage to nucleotide bases, base mismatches, and bulky DNA lesions that distort the DNA double helix. Base excision repair (BER) mends small non-distorting DNA lesions such as oxidized DNA bases (Wallace, 2014), while nucleotide excision repair (NER) corrects bulky lesions such UV-induced pyrimidine dimers (Schärer, 2013). BER is mediated by DNA glycosylases that cleave and remove the damaged bases at the lesion. A correct base is then inserted by specialized DNA polymerases (Wallace, 2014). In NER, a 25–30 nucleotide patch of ssDNA containing the bulky lesion is excised from the double strand helix before specialized DNA polymerases fill in the ssDNA gap (Schärer, 2013). Mismatch repair (MMR) proofreads the newly replicated DNA to identify mis-incorporated bases (Liu D. et al., 2017). When a mismatch is identified, the newly synthesized strand is nicked and resected creating a patch of exposed ssDNA for fill in by a high-fidelity DNA polymerase.

### Essentiality of DNA Repair During Embryogenesis

Not all DNA repair activities are essential for early development (Supplementary Table S1). The most striking example is the dichotomy between the major DSB repair pathways. While many HDR factors are essential, the core c-NHEJ factors are either dispensable for development [*Ku70* (Ouyang et al., 1997), *Ku80* (Nussenzweig et al., 1996; Gu et al., 1997), *Prkdc* (DNA-PKcs) (Kurimasa et al., 1999), *Nhej1* (XLF) (Li et al., 2008)] or only required for organogenesis [*Lig4* (Frank et al., 1998), *Xrcc4* (Dickinson et al., 2016)]. Some alt-NHEJ components are required for early development. Alt-NHEJ is promoted by the polymerase Pol θ which primes the DNA ends for repair by removing RPA from exposed ssDNA regions (Kent et al., 2015; Mateos-Gomez et al., 2017). Alt-NHEJ is also dependent on PARP-1, XRCC1 and LIG3 (Audebert et al., 2004), with PARP-1 having a critical role in synapsis, and XRCC1 and LIG3 mediating DNA ligation (Audebert et al., 2004). While *Lig3* and *Xrcc1* knockout embryos die during gastrulation (Tebbs et al., 1999; Puebla-Osorio et al., 2006), both genes encode factors that have functions in other DNA metabolic activities outside EJ (Wallace, 2014). Conversely *Pol θ* knockout mice are viable (Shima et al., 2004; Masuda et al., 2005), as are *Parp1* knockouts (Wang et al., 1995), suggesting that alt-NHEJ is not essential for embryogenesis.

Preference for HDR over NHEJ is further evident in mechanistic studies of DDR engagement within embryonic stem cells (Tichy et al., 2010). Following DSB induction, ATM-dependent γ-H2AX phosphorylation at the break locus recruits the scaffolding protein MDC1 (Stewart et al., 2003). MDC1 subsequently recruits the ubiquitin ligases RNF8 and RNF168 that signal downstream recruitment of 53BP1 (Jackson and Durocher, 2013). 53BP1 then establishes a physical domain at the repair locus in coordination with RIF1 to inhibit end resection and HDR in favor of c-NHEJ (Chapman et al., 2013; Zimmermann et al., 2013; Lou et al., 2019; Ochs et al., 2019). Notably, many factors that promote c-NHEJ are dispensable for embryonic development, including *Atm* (Barlow et al., 1996; Xu et al., 1996), *H2ax* (Celeste et al., 2002), *Mdc1* (Lou et al., 2006), *Rnf8* (Valnegri et al., 2017), *Rfn168* (Zong et al., 2019), and *Trp53bp1* (53BP1) (Ward et al., 2003). Additionally, multiple reports demonstrate that 53BP1 does not localize to DSB foci in the ICM of preimplantation embryos or mESCs grown in culture (Ziegler-Birling et al., 2009; Kafer et al., 2016). *In vivo*, 53BP1 localizes to damage in E5.5 embryos, but only in the epiblast cells and not the endoderm or trophoblast cells (Laurent and Blasi, 2015). While *Trp53bp1* expression is unchanged in early development, *Rnf168* expression is limited prior to the epiblast stage which may explain differential engagement of DDR pathways in pre- and peri-implantation cells (Laurent and Blasi, 2015).

## Genes Essential for Preimplantation Development

Consistent with a greater tolerance of genome instability in preimplantation cells, only 10 knockouts of genome stability factors that we reviewed were lethal during preimplantation (Table 1 and Supplementary Table S1). Factors encoded by many of these preimplantation essential genes also possessed critical functions outside DDR or repair. Additionally, preimplantation embryos commonly survive to the morula stage even if a targeted gene is essential for viability. This is because embryonic genomes are not immediately active following fertilization while the inherited maternal mRNA guides protein translation (Jukam et al., 2017).

### Multifunctional Factors Essential for Preimplantation Development

Of the 10 genome stability factors required for preimplantation development, only *Ercc2* deletion did not permit morula generation. *Ercc2*^–/–^ embryos fail to develop past the 2-cell stage *in vitro* and there is no evidence of embryo implantation *in vivo* (de Boer et al., 1998). *Ercc2* encodes the XPD helicase that functions both in NER and as a core component of the general transcription factor IIH (TFIIH) complex. TFIIH opens DNA and anchors the cdk-activating kinase (CAK) complex to facilitate transcription (Egly, 2001). Given that *Ercc2* knockouts are compromised during embryonic genome activation (EGA), a reasonable conclusion is that preimplantation lethality is associated with a loss of XPD transcriptional activity. Likewise, *Xab2* is a TFIIH and NER component and *Xab2* deletion is lethal prior to the blastocyst stage (Yonemasu et al., 2005). However, *Xab2* null embryos survive past the 2-cell stage suggesting *Xab2* is not required for EGA.

*Pcna* and *Rpa1* both function in multiple genome maintenance pathways and deleting either gene confers preimplantation lethality (Wang et al., 2005; Roa et al., 2008). PCNA (proliferating cell nuclear antigen) forms a homotrimeric sliding clamp that encircles DNA. The protein clamp travels with the replication fork to promote replicative polymerase processivity and mediate diverse functions in DNA replication and the replication stress response (Mailand et al., 2013). PCNA also functions in NER and MMR (Strzalka and Ziemienowicz, 2011). *Rpa1* (Replication Protein A 1) codes for the 70 kDa subunit of the heterotrimeric RPA ssDNA binding complex that participates in replication, but which also functions in ATR activation, replication fork repair, HDR and NER (Bhat and Cortez, 2018). Analysis of *Pcna* mRNA showed a significant reduction of maternal transcripts from the zygote to the 2-cell stage consistent with the observed early embryonic lethality (Hamatani et al., 2004; Roa et al., 2008; Bult et al., 2019). *Rpa1* null embryos form blastocysts at E3.5 that are smaller than their heterozygous littermates and which fail in blastocyst outgrowth assays, indicating *Rpa1* is required for trophectoderm and ICM growth (Wang et al., 2005). While both *Rpa1* and *Pcna* function in diverse aspects of genome maintenance, both genes are also required for DNA replication. Preimplantation failure of *Pcna* or *Rpa1* null embryos likely stems from an inability to polymerize nascent DNA coupled with simultaneous attenuation of multiple DNA repair pathways.

Two additional genes classified as DNA repair factors in the MGI-GO database are also essential during preimplantation. *Nop53* encodes a nucleolar protein with suggested roles regulating p53, DNA repair, and the cellular response to mitochondrial stress (Lee et al., 2012; Yoon et al., 2014). *Nop53* null embryos fail to develop past the blastocyst stage, while *Nop53*^–/–^ mESCs are viable, suggesting that *Nop53* functions in morula to blastocyst maturation (Sasaki et al., 2011). In somatic cells, *Pot1a* encodes a telomere specific ssDNA binding protein that suppresses ATR and p53 activation by the chromosome end (Hockemeyer et al., 2005, 2006; Denchi and de Lange, 2007). Loss of *Pot1a* likely confers robust ATR activation which explains the failure of *Pot1a*^–/–^ embryos to form an ICM (Hockemeyer et al., 2006).

### Cell Cycle and Checkpoint Factors Essential for Preimplantation Viability

In the event of replication stress or DSBs, checkpoints slow the cell cycle to provide time for DNA repair. Deletion of several genes that encode checkpoint regulatory factors confer preimplantation embryo failure at the morula stage. These include *Wee1* (Tominaga et al., 2006), *Cdk1* (Diril et al., 2012), and *Plk1* (Lu et al., 2008; Wachowicz et al., 2016). CDK1 promotes the G2/M transition through interaction with cyclin B, and CDK1-cyclin B activity is regulated by WEE1 (Harvey et al., 2005; Gavet and Pines, 2010). ATR and CHK1 activate the G2/M checkpoint by stimulating WEE1 to inhibit CDK1 (O’Connell et al., 1997; Nigg, 2001). Polo-like kinase 1 (PLK1) controls numerous mitotic activities, participates in the G2/M transition, may function in DNA replication, and is implicated in HDR through phosphorylation of RAD51 (Watanabe et al., 2004; Mandal and Strebhardt, 2013; Yata et al., 2014; Pintard and Archambault, 2018). *Dtl* is also required for preimplantation development (Liu et al., 2007). *Dtl* encodes a component of the CUL4A-DDB1 E3-ubiquitin ligase complex that degrades the replication licensing factor CDT1 to stimulate replication and S phase progression (Nishitani et al., 2001; Li and Blow, 2005). Because these essential cell cycle genes function in DDR-regulated checkpoints and normal cell cycle transitions, the mechanism of embryonic lethality following their deletion likely involves deregulation of both the DDR and normal cell cycles.

## Genes Essential for Peri-Implantation Development

Peri-implantation is the period of embryonic development most associated with lethality following deletion of genome stability factors. Within the MGI-GO derived gene list, it stands out that 54 of the 62 genes essential for peri-implantation development have direct or indirect functions in DNA replication, the replication stress response, and/or HDR (Table 2 and Supplementary Table S1). Below we focus specifically on these pathways.

### Genes Regulating Replication Initiation, DNA Polymerization, and the Replication Stress Response Are Essential for Peri-Implantation Development

Because of the rate of cell proliferation in peri-implantation embryos, it is perhaps unsurprising that factors which regulate the G1/S transition and initiate DNA replication are essential for peri-implantation development. This includes *Cdc7* (Kim et al., 2002), *Cdc45* (Yoshida et al., 2001), *Cdk7* (Ganuza et al., 2012), *Mnat1* (Rossi et al., 2001), *Gins4* (Mohri et al., 2013), *Cul4A* (Li et al., 2002), and *Ddb1* (Cang et al., 2006). Additionally, the *Pold3* subunit of the replicative polymerase *Polδ* is required for peri-implantation development (Uchimura et al., 2009; Zhou et al., 2018), as is *Fen1*, which plays a central role in processing Okazaki fragments on the lagging strand of DNA synthesis (Larsen et al., 2003). Lethality following deletion of most of these genes occurs on or near E4.5 and is associated with proliferative failure within the ICM after blastocyst formation but prior to gastrulation. A likely explanation is that peri-implantation development is rapidly compromised when fundamental functions necessary for cellular proliferation and/or DNA replication are dysregulated.

Even in the absence of exogenous threats to replication, the exceptionally fast cell cycles within the developing epiblast are expected to confer intrinsic replication stress. For example, blastocyst derived mESCs display replication stress in unperturbed cultures *in vitro* (Ahuja et al., 2016). mESCs appear to manage this replication stress by effectively coupling replication and repair activities to facilitate near continuous DNA synthesis punctuated by brief G1 and M phases (Ahuja et al., 2016). Epiblast cell cycles *in vivo* are more rapid than cell cycles in cultured mESCs (Snow, 1977; Mohammed et al., 2017) suggesting that a similar reliance on efficient coupling of DNA replication and the repair activity is required to manage replication stress in peri-implantation embryos.

The replication stress response begins when ATR/ATRIP localizes to RPA-coated ssDNA, and ATR is activated through TOPBP1 and the RAD9-RAD1-HUS1 (9-1-1) protein complex (Kumagai et al., 2006; Navadgi-Patil and Burgers, 2009; Choi et al., 2010). 9-1-1 is a sliding clamp loaded onto DNA (Bermudez et al., 2003). TOPBP1 bridges ATR/ATRIP and 9-1-1, thereby stabilizing ATR at the fork. Once stabilized at the site of replication stress, ATR regulates downstream replication stress repair and cell cycle arrest via CHK1 kinase and CDC25A (Liu et al., 2000; Xiao et al., 2003; Delacroix et al., 2007). TIMELESS, a component of the replication fork protection complex (Kemp et al., 2010; Couch et al., 2013; Buisson et al., 2017), and the ubiquitin ligase PRP19 (*Prpf19*) (Maréchal et al., 2014), also function in ATR and CHK1 activation. Embryos null for *Atr* (Brown and Baltimore, 2000; de Klein et al., 2000; Murga et al., 2009), *Topbp1* (Jeon et al., 2011), *Hus1* (Weiss et al., 2000), *Chek1* (CHK1) (Takai et al., 2000; Zaugg et al., 2007), *Cdc25A* (Ray et al., 2007), *Timeless* (Gotter et al., 2000), and *Prpf19* (Fortschegger et al., 2007) are all compromised during peri-implantation.

In somatic tissues, ATR or CHK1 inhibition coupled with replication stress induces replication catastrophe and S-phase apoptosis (Myers et al., 2009; Toledo et al., 2013; van Harten et al., 2019). Similarly, *Atr* null embryos form blastocysts that *in vitro* display widespread apoptosis within the ICM but not in the trophoblast, and which are eventually compromised by day three of culture (Brown and Baltimore, 2000; de Klein et al., 2000; Murga et al., 2009). ATR activity is therefore essential in the pluripotent and rapidly proliferating epiblast cells that will become the embryo proper, but not in the multipotent placental precursors. *Chek1* null embryos also form blastocysts, but unlike *Atr* null embryos, are unable to attach to the culture vessels in outgrowth experiments (Takai et al., 2000). The more severe embryonic response to a loss of CHK1 activity compared to ATR inhibition is consistent with observations in somatic cells (Buisson et al., 2015).

### HDR Genes Are Essential for Peri-Implantation Development

Common HDR factors manipulate DNA substrates at two-ended DNA breaks and stressed replication forks (Ait Saada et al., 2018). During replication stress, this entails regressing and protecting stalled forks in 4-way DNA structures to facilitate repair (Quinet et al., 2017). HDR factors essential for peri-implantation that function in canonical DSB repair and replication fork protection include: *Brca1* (Hakem et al., 1996; Ludwig et al., 1997; Taglialatela et al., 2017), *Brca2* (Sharan et al., 1997; Lemaçon et al., 2017; Mijic et al., 2017), *Rad51* (Lim and Hasty, 1996; Tsuzuki et al., 1996; Zellweger et al., 2015), *Bard1* (McCarthy et al., 2003; Shakya et al., 2008; Daza-Martin et al., 2019), *Palb2* (Bowman-Colin et al., 2013), and *Rad51C* (Kuznetsov et al., 2009; Somyajit et al., 2015). Similarly, common factors promote resection during HDR repair of canonical two-ended DSBs and at stalled replication forks (Ait Saada et al., 2018). This includes the essential peri-implantation genes: *Ctip* (Chen et al., 2005; Przetocka et al., 2018); *Ptip* (Cho et al., 2003; Ray Chaudhuri et al., 2016); *Dna2* (Lin et al., 2013; Thangavel et al., 2015), and the MRN complex [*Mre11*, *Rad50*, and *Nbs1* (Xiao and Weaver, 1997; Luo et al., 1999; Zhu et al., 2001; Theunissen et al., 2003; Buis et al., 2008; Schlacher et al., 2011).

Homology directed repair activity in replication fork remodeling and protection is an emerging topic in the DNA repair field, and it remains unclear which HDR functions are essential for cell viability. However, lethality in *Brca2* null mESCs is rescued when fork protection is facilitated by inhibiting PARP activity to protect replication forks from MRE11 nuclease (Ding et al., 2016). For BRCA2, fork protection may be the essential function. *In vivo*, deleting factors that participate in both canonical HDR-dependent DSB repair and fork remodeling or resection, typically confers reduced cell proliferation and embryo failure during gastrulation. For example, *Brca1*^–/–^ embryos are severely malformed with an underdeveloped pro-amniotic cavity and mesoderm (Hakem et al., 1996; Ludwig et al., 1997). Concordantly, *Brca1* null embryos display a significant reduction of DNA synthesis by E6.5, increased p21 [a factor induced by p53 transactivation to mediate cell cycle arrest (Georgakilas et al., 2017)], and a high incidence of G1 arrested cells (Hakem et al., 1996). Similarly, *Rad51* null embryos suffer from reduced proliferation and begin to degenerate during gastrulation by E7.5, but development can be extended to E9.5 in *TP53/Rad51* double knockouts (Lim and Hasty, 1996). Loss of HDR factors therefore appears to primarily confer proliferative failure through p53-induced growth arrest and subsequently through accumulated genome instability if p53 function is compromised.

### Additional Peri-Implantation Genes Linked to HDR or Replication

Peri-implantation development additionally requires several genes that are functionally linked to HDR or replication stress mitigation. Such genes include *Apex1* (Xanthoudakis et al., 1996), *Rnaseh2B* (Hiller et al., 2012), *Rif1* (Chapman et al., 2013), *Blm* (Chester et al., 1998), *Rev3L* (Bemark et al., 2000), *Usp7* (Kon et al., 2010), and *Ino80* (Min et al., 2013). *Apex1* does not directly function in replication, but is critical for the repair of abasic DNA sites that if left unresolved confer replication stress (Boiteux and Guillet, 2004). Similarly, *Rnaseh2B* resolves RNA within RNA-DNA hybrids, including mis-incorporated ribonucleotides, which if not resolved can impede replication (Pizzi et al., 2015). In addition to functioning in DSB repair pathway choice, *Rif1* also coordinates replication timing (Yamazaki et al., 2012; Foti et al., 2016). *Blm* encodes a helicase that functions in homologous recombination, the replication stress response (Davies et al., 2007; Machwe et al., 2011), promotes replication at telomeres (Barefield and Karlseder, 2012), and participates in a specialized form of break induced replication at chromosome ends (Sobinoff et al., 2017). *Rev3L* encodes the catalytic subunit of POL ζ which facilitates translesion synthesis to enable replication forks to negotiate lesions within DNA without stalling (Bhat et al., 2013). *Usp7* is a deubiquitylating enzyme that promotes efficient DNA replication (Lecona et al., 2016) and *Ino80* encodes a chromatin remodeling factor that functions in both replication and HDR (Poli et al., 2017).

## Maintaining Genome Stability During Peri-Implantation Development

While HDR and replication associated genes are not the only genome stability factors required for peri-implantation development, their predominance on the list of genes essential for this developmental window is compelling. Conditions enabling *in vitro* culture of peri-implantation murine epiblast stem cells are now established (Brons et al., 2007; Tesar et al., 2007). However, there are no published studies focusing on genome maintenance in isolated epiblast cells. It is thus necessary to extrapolate from studies of other rapidly dividing cells why HDR and replication factors may be essential during peri-implantation development.

### Replication Stress in Peri-Implantation Cells

The fastest cell cycles in the lifetime of a mouse are likely during the brief window of epiblast development. Outside of the embryo, rapid proliferation contributes to replication stress in hematopoietic and cancerous tissues by robbing cells of the time to properly execute DNA polymerization (Pilzecker et al., 2017). For example, during a somatic G1-phase replication is facilitated by increasing the abundance of MCM proteins and loading these factors at replication origins in preparation for S-phase (Boos et al., 2012). Late G1 cells also upregulate dNTP production for the oncoming burst of DNA synthesis (Hirschi et al., 2010; Dick and Rubin, 2013). When origins are not established properly, or dNTP production is dysregulated, cancer and aging hematopoietic cells are susceptible to replication stress (Flach et al., 2014; Aird and Zhang, 2015; Alvarez et al., 2015; Garzon et al., 2017).

Pluripotent cells in the early embryo display almost non-existent G1 phases and appear to use unique mechanisms to ensure S-phase progression is not impeded. For example, human ESCs counter G1 brevity by loading MCM proteins faster than cultured non-pluripotent cells (Matson et al., 2017). Retinoblastoma protein is also constitutively hyperphosphorylated in mouse and human ESCs (Conklin et al., 2012; Ter Huurne et al., 2017), which maintains high E2F transactivation to promote unperturbed dNTP synthesis (Hirschi et al., 2010; Dick and Rubin, 2013). To ensure rapid S-phase progression, mouse ESCs also license more dormant origins than non-embryonic progenitor cells (Ge et al., 2015). This increases the ability of ESCs to complete replication during a short S phase (Courtot et al., 2018). ESCs also demonstrate an increased ability to restart stalled replication forks (Ahuja et al., 2016; Zhao et al., 2018). Human induced pluripotent stem cells (iPSC) also license replication origins rapidly in their abbreviated G1 phase (Matson et al., 2017), and dormant origins are critical to maintain rapid proliferation in hematopoietic stem cell pools (Alvarez et al., 2015). Although not yet directly tested, we anticipate that epiblast cells employ similar countermeasures to expedite DNA synthesis.

Despite these efforts, human and mouse ESCs display endogenous replication stress in unperturbed cultures (Ahuja et al., 2016; Lamm et al., 2016). It is not clear why replication is stressed in pluripotent cells, however in proliferating cancer cells transcription commonly drives replication stress through collisions between RNA and DNA polymerases, or persistent RNA/DNA hybrid molecules termed R-loops (Crossley et al., 2019). Primary somatic cells mitigate transcription and R-loop interference by temporally coordinating transcription and replication during S-phase (Meryet-Figuiere et al., 2014). It is probable that peri-implantation epiblast cells facilitate rapid genome duplication by simultaneously engaging large numbers of origins. Such activity could increase the probability of replisome and transcriptome collisions and/or R-loop induced replication stress.

Under-replication due to endogenous stress is described in mESCs and human iPSCs (Ahuja et al., 2016; Vallabhaneni et al., 2018). Lengthening the G1 phase reduces replication defects in mESCs consistent with the notion that replication stress is driven by rushed cell cycles (Ahuja et al., 2016). Pluripotent cells are reported to have enhanced DNA repair capacities and it is possible this translates to highly effectively management of inefficient replication in embryos (Maynard et al., 2008; Luo et al., 2012). Alternatively, pluripotent blastocyst cells persist for a limited number of cell divisions *in vivo*, and it is feasible that pre-implantation embryos simply tolerate under-replication for this brief period. Tolerance of replication defects is consistent with observations that preimplantation embryonic cells are more resistant to genomic insult than peri-implantation cells. Compromising the replication stress response should confer similar DDR activation, chromosomal segregation errors, and aneuploidy in both pre- and peri-implantation cells. However, cells with supernumerary chromosomes persist in the blastocyst before elimination during peri-implantation (Bolton et al., 2016). This may explain why many genome stability factors become essential specifically during peri-implantation development. *In vivo*, activating p53 through deletion of its negative regulator *Mdm2* leads to lethality during peri-implantation (Jones et al., 1995). This suggests that p53 signaling during peri-implantation is a conduit to remove genomically unstable cells *in vivo*.

Additionally, it is important to recognize that genome stability factors may possesses cryptic essential functions during early development. For example, PARP1 is DDR factor involved in multiple aspects of genome integrity (Ray Chaudhuri and Nussenzweig, 2017). However, in mESCs, *Parp1* has roles preventing the *trans*-differentiation of extraembryonic trophoblast cells (Nozaki et al., 1999; Hemberger et al., 2003). This pluripotency-specific function is linked to the DNA binding ability of PARP1 which confers an epigenetic-like regulation of pluripotency (Roper et al., 2014). Additionally, pluripotency-specific repair factors may provide essential functions in early development. The Filia-Floped protein complex is abundantly expressed in pluripotent but not somatic cells, and is suggested to increase the abundance of essential repair factors including BLM and promote ATR activation to encourage fork restart (Zhao et al., 2015; Zhao et al., 2018; Zheng, 2020).

### Essential Roles for HDR Factors During Peri-Implantation

It is possible the canonical G1-phase c-NHEJ DSB repair pathway is dispensable during peri-implantation because cells rapidly transition through their brief G1 phase and engage alt-NHEJ or HDR in S-phase. Conversely, c- and alt-NHEJ remain active in HDR-deficient embryos, and in principle EJ functions could provide redundant DSB repair activity. An explanation why c- or alt-NHEJ cannot rescue HDR during peri-implantation may stem from the diversity of substrates repaired by HDR. While two-ended DSBs are readily repaired by EJ, collapsed replication forks present as one-ended DNA breaks which c- or alt-NHEJ cannot process (Feng and Jasin, 2017; Scully et al., 2019). One-ended breaks, however, can be repaired through a specialized HDR mechanism termed break-induced replication (BIR). During BIR, the exposed DNA end is resected, and following strand-invasion to form a displacement loop on the sister chromatid, DNA is polymerized from the invaded strand in a conservative manner (Kramara et al., 2018). While BIR is commonly studied in yeast, the analogous mechanism in vertebrates remains poorly defined. Notwithstanding, *Blm* promotes BIR at chromosome ends in human cancer cells that maintain telomere length through the alternative lengthening of telomeres mechanism suggesting that *Blm* may promote BIR elsewhere in the genome following replication stress (Sobinoff et al., 2017). Additionally, *Pold3* is also implicated in vertebrate BIR (Costantino et al., 2014). Conversely, the non-essential gene *Rad52* also functions in vertebrate BIR casting doubt on the requirement for BIR in early development and indicating the need for future studies (Sotiriou et al., 2016).

Multiple HDR factors essential for peri-implantation also play central roles in replication stress management. This includes HDR factors that regress, stabilize, or resect stalled replication forks including *Brca1/2, Bard1, Rad51* and *Rad51C*, *Ctip, Ptip, Dna2*, and genes encoding the MRN complex. Interestingly, the helicases and translocases that promote fork regression, including *Smarcal1*, are not essential for peri-implantation (Baradaran-Heravi et al., 2012). This may reflect redundancy in the mechanisms that drive formation of 4-strand structures at stalled replication forks (Quinet et al., 2017). Given the essentiality of the replication stress response for epiblast development, it stands to reason that replication fork remodeling and protective functions encoded by HDR factors play a role in the viability of peri-implantation embryos. Failure to facilitate these activities would confer genome instability, chromosome segregation errors, and molecular outcomes consistent with unrepaired replication stress. As described above, this would activate p53 surveillance systems during peri-implantation. In the future it will be interesting to determine which specific HDR functions are essential for embryogenesis.

## Conclusion and Future Directions

While most DDR and repair factors are non-essential, this does not exclude non-essential genome stability pathways from playing an important role in healthy development. Deletion of non-essential DDR and repair genes commonly results in the birth of live pups exhibiting a wide array of deleterious phenotypes (Supplementary Table S1). Additionally, cancer predisposition and a reduced lifespan are common in mice lacking non-essential genome stability factors (Supplementary Table S1). The difference between essential and non-essential genome stability genes is likely derived from the necessity of the targeted repair pathway to resolve lethal genome instability that arises during a specific window of development.

The critical genome stability pathways required for cell survival are determined by the type of genomic lesions the cell encounters and when in the cell cycle those lesions arise. Early embryonic development is largely protected from exogenous influence. Preimplantation embryos are wholly contained, and maternal blood supply begins at E9.5 (Behringer et al., 2014). The critical early stages of development likely benefit from sequestration from external threats to genome stability. We suggest the imminent threat to genome stability in early development stems from the need to mitigate endogenous replication stress within the epiblast to sustain rapid cell cycles. If left unresolved, replication stress drives genome instability, chromosome segregation errors, growth arrest and/or cell death. Dwindling cell numbers of epiblast cells that fail to effectively replicate their genome will progressively lead to embryo compromise. This premise is supported by the timing of embryo failure associated with deletion of essential DDR and repair genes. Consistent with systemic proliferative failure, deletion of factors required for basal DNA replication, or up-stream signaling in the replication stress response, typically induce an early embryonic demise (E5.0 or before). Whereas deleting HDR factors induces death at a subsequent time (E5.5 or later), potentially as the additive outcome of progressive genome instability.

Another caveat of reports reviewed here is that many were pioneering studies that utilized the newly developed technology of gene targeting in mice. In the intervening decades, our understanding of early development and experimental capability has blossomed. Development of new stem cell culture technologies now enables mechanistic study of peri-implantation development within three-dimensional gastrula structures *in vitro*. Co-culture of ICM derived ESCs, trophectoderm derived trophoblast stem cells, and extraembryonic endoderm stem cells results in the spontaneous self-assembly of a structure remarkably like the gastrulating embryo. These structures, termed “ETX embryos” will enable directed gene deletion within specific embryonic cell types (Sozen et al., 2018). Coupled with precise mechanistic investigations, ETX embryo models will reveal new insights on the mechanisms of cell compromise during embryogenesis. It will be exciting to learn in the coming years why a limited number of DDR and repair pathways are essential for development, and the underlying reasons for their importance within the peri-implantation embryo.

## Data Availability Statement

MGI-GO designations discussed in this review can be found at the following. DNA damage checkpoint: http://www.informatics.jax.org/go/term/GO:0000077; nucleotide excision repair: http://www.informatics.jax.org/go/term/GO:0006289; mismatch re- pair: http://www.informatics.jax.org/go/term/GO:0006298; base excision repair: http://www.informatics.jax.org/go/term/GO:0006284; DSB repair via homologous recombination: http://www.informatics.jax.org/go/term/GO:0000724; DSB repair via non-homologous end joining: http://www.informatics.jax.org/go/term/GO:0006303.

## Author Contributions

GK and AC conceived of the review manuscript. GK compiled the information presented in Tables 1, 2 and Supplementary Table S1 from the MGI-GO database as indicated. GK made the figures. GK and AC composed the manuscript.

## Conflict of Interest

The authors declare that the research was conducted in the absence of any commercial or financial relationships that could be construed as a potential conflict of interest.

## References

[B1] Acloque H. Adams M. S. Fishwick K. Bronner-Fraser M. Nieto M. A. (2009). Epithelial-mesenchymal transitions: the importance of changing cell state in development and disease. *J. Clin. Invest.* 119 1438–1449. 10.1172/JCI38019 19487820 PMC2689100

[B2] Affar El B. Gay F. Shi Y. Liu H. Huarte M. Wu S. (2006). Essential dosage-dependent functions of the transcription factor yin yang 1 in late embryonic development and cell cycle progression. *Mol. Cell. Biol.* 26 3565–3581. 10.1128/MCB.26.9.3565-3581.2006 16611997 PMC1447422

[B3] Afzal S. Hao Z. Itsumi M. Abouelkheer Y. Brenner D. Gao Y. (2015). Autophagy-independent functions of UVRAG are essential for peripheral naive T-cell homeostasis. *Proc. Natl. Acad. Sci. U.S.A.* 112 1119–1124. 10.1073/pnas.1423588112 25583492 PMC4313859

[B4] Ahuja A. K. Jodkowska K. Teloni F. Bizard A. H. Zellweger R. Herrador R. (2016). A short G1 phase imposes constitutive replication stress and fork remodelling in mouse embryonic stem cells. *Nat. Commun.* 7:10660. 10.1038/ncomms10660 26876348 PMC4756311

[B5] Aird K. M. Zhang R. (2015). Nucleotide metabolism, oncogene-induced senescence and cancer. *Cancer Lett.* 356 204–210. 10.1016/j.canlet.2014.01.017 24486217 PMC4115046

[B6] Ait Saada A. Lambert S. A. E. Carr A. M. (2018). Preserving replication fork integrity and competence via the homologous recombination pathway. *DNA Repair* 71 135–147. 10.1016/j.dnarep.2018.08.017 30220600 PMC6219450

[B7] Akdemir K. C. Jain A. K. Allton K. Aronow B. Xu X. Cooney A. J. (2014). Genome-wide profiling reveals stimulus-specific functions of p53 during differentiation and DNA damage of human embryonic stem cells. *Nucleic Acids Res.* 42 205–223. 10.1093/nar/gkt866 24078252 PMC3874181

[B8] Ali A. A. E. Timinszky G. Arribas-Bosacoma R. Kozlowski M. Hassa P. O. Hassler M. (2012). The zinc-finger domains of PARP1 cooperate to recognize DNA strand breaks. *Nat. Struct. Mol. Biol.* 19 685–692. 10.1038/nsmb.2335 22683995 PMC4826610

[B9] Alvarez S. Diaz M. Flach J. Rodriguez-Acebes S. Lopez-Contreras A. J. Martinez D. (2015). Replication stress caused by low MCM expression limits fetal erythropoiesis and hematopoietic stem cell functionality. *Nat. Commun.* 6:8548. 10.1038/ncomms9548 26456157 PMC4608254

[B10] Andressoo J. O. Weeda G. De Wit J. Mitchell J. R. Beems R. B. Van Steeg H. (2009). An Xpb mouse model for combined xeroderma pigmentosum and cockayne syndrome reveals progeroid features upon further attenuation of DNA repair. *Mol. Cell. Biol.* 29 1276–1290. 10.1128/MCB.01229-08 19114557 PMC2643825

[B11] Artus J. Vandormael-Pournin S. Frodin M. Nacerddine K. Babinet C. Cohen-Tannoudji M. (2005). Impaired mitotic progression and preimplantation lethality in mice lacking OMCG1, a new evolutionarily conserved nuclear protein. *Mol. Cell. Biol.* 25 6289–6302. 10.1128/MCB.25.14.6289-6302.2005 15988037 PMC1168835

[B12] Audebert M. Salles B. Calsou P. (2004). Involvement of poly(ADP-ribose) polymerase-1 and XRCC1/DNA ligase III in an alternative route for DNA double-strand breaks rejoining. *J. Biol. Chem.* 279 55117–55126. 10.1074/jbc.M404524200 15498778

[B13] Banin S. Moyal L. Shieh S. Taya Y. Anderson C. W. Chessa L. (1998). Enhanced phosphorylation of p53 by ATM in response to DNA damage. *Science* 281 1674–1677. 10.1126/science.281.5383.1674 9733514

[B14] Baradaran-Heravi A. Cho K. S. Tolhuis B. Sanyal M. Morozova O. Morimoto M. (2012). Penetrance of biallelic SMARCAL1 mutations is associated with environmental and genetic disturbances of gene expression. *Hum. Mol. Genet.* 21 2572–2587. 10.1093/hmg/dds083 22378147 PMC3349428

[B15] Barefield C. Karlseder J. (2012). The BLM helicase contributes to telomere maintenance through processing of late-replicating intermediate structures. *Nucleic Acids Res.* 40 7358–7367. 10.1093/nar/gks407 22576367 PMC3424559

[B16] Barlow C. Hirotsune S. Paylor R. Liyanage M. Eckhaus M. Collins F. (1996). Atm-deficient mice: a paradigm of ataxia telangiectasia. *Cell* 86 159–171. 10.1016/s0092-8674(00)80086-0 8689683

[B17] Bass T. E. Luzwick J. W. Kavanaugh G. Carroll C. Dungrawala H. Glick G. G. (2016). ETAA1 acts at stalled replication forks to maintain genome integrity. *Nat. Cell Biol.* 18 1185–1195. 10.1038/ncb3415 27723720 PMC5245861

[B18] Behringer R. Gertsenstein M. Nagy K. V. Nagy A. (2014). *Manipulating the Mouse Embryo.* Cold Spring Harbor, NY: Cold Spring Harbor Laboratory Press.

[B19] Bemark M. Khamlichi A. A. Davies S. L. Neuberger M. S. (2000). Disruption of mouse polymerase zeta (Rev3) leads to embryonic lethality and impairs blastocyst development in vitro. *Curr. Biol.* 10 1213–1216. 10.1016/s0960-9822(00)00724-7 11050391

[B20] Bermudez V. P. Lindsey-Boltz L. A. Cesare A. J. Maniwa Y. Griffith J. D. Hurwitz J. (2003). Loading of the human 9-1-1 checkpoint complex onto DNA by the checkpoint clamp loader hRad17-replication factor C complex *in vitro*. *Proc. Natl. Acad. Sci. U.S.A.* 100, 1633–1638. 10.1073/pnas.0437927100 12578958 PMC149884

[B21] Bhat A. Andersen P. L. Qin Z. Xiao W. (2013). Rev3, the catalytic subunit of Polzeta, is required for maintaining fragile site stability in human cells. *Nucleic Acids Res.* 41 2328–2339. 10.1093/nar/gks1442 23303771 PMC3575803

[B22] Bhat K. P. Cortez D. (2018). RPA and RAD51: fork reversal, fork protection, and genome stability. *Nat. Struct. Mol. Biol.* 25 1–8. 10.1038/s41594-018-0075-z 29807999 PMC6006513

[B23] Bleichert F. (2019). Mechanisms of replication origin licensing: a structural perspective. *Curr. Opin. Struct. Biol.* 59 195–204. 10.1016/j.sbi.2019.08.007 31630057

[B24] Boiteux S. Guillet M. (2004). Abasic sites in DNA: repair and biological consequences in Saccharomyces cerevisiae. *DNA Repair.* 3 1–12. 10.1016/j.dnarep.2003.10.002 14697754

[B25] Bolton H. Graham S. J. L. Van Der Aa N. Kumar P. Theunis K. Fernandez Gallardo E. (2016). Mouse model of chromosome mosaicism reveals lineage-specific depletion of aneuploid cells and normal developmental potential. *Nat. Commun.* 7:11165. 10.1038/ncomms11165 27021558 PMC4820631

[B26] Boos D. Frigola J. Diffley J. F. (2012). Activation of the replicative DNA helicase: breaking up is hard to do. *Curr. Opin. Cell Biol.* 24 423–430. 10.1016/j.ceb.2012.01.011 22424671

[B27] Bowman-Colin C. Xia B. Bunting S. Klijn C. Drost R. Bouwman P. (2013). Palb2 synergizes with Trp53 to suppress mammary tumor formation in a model of inherited breast cancer. *Proc. Natl. Acad. Sci. U.S.A.* 110 8632–8637. 10.1073/pnas.1305362110 23657012 PMC3666744

[B28] Brons I. G. Smithers L. E. Trotter M. W. Rugg-Gunn P. Sun B. Chuva De Sousa Lopes S. M. (2007). Derivation of pluripotent epiblast stem cells from mammalian embryos. *Nature* 448 191–195. 10.1038/nature05950 17597762

[B29] Brown E. J. Baltimore D. (2000). ATR disruption leads to chromosomal fragmentation and early embryonic lethality. *Genes Dev.* 14 397–402. 10691732 PMC316378

[B30] Buis J. Wu Y. Deng Y. Leddon J. Westfield G. Eckersdorff M. (2008). Mre11 nuclease activity has essential roles in DNA repair and genomic stability distinct from ATM activation. *Cell* 135 85–96. 10.1016/j.cell.2008.08.015 18854157 PMC2645868

[B31] Buisson R. Boisvert J. L. Benes C. H. Zou L. (2015). Distinct but concerted roles of ATR, DNA-PK, and Chk1 in countering replication stress during S Phase. *Mol. Cell* 59 1011–1024. 10.1016/j.molcel.2015.07.029 26365377 PMC4575890

[B32] Buisson R. Niraj J. Rodrigue A. Ho C. K. Kreuzer J. Foo T. K. (2017). Coupling of Homologous Recombination and the Checkpoint by ATR. *Mol. Cell* 65 336–346. 10.1016/j.molcel.2016.12.007 28089683 PMC5496772

[B33] Bult C. J. Blake J. A. Smith C. L. Kadin J. A. Richardson J. E. Mouse Genome Database G. (2019). Mouse Genome Database (MGD) 2019. *Nucleic Acids Res.* 47 D801–D806. 10.1093/nar/gky1056 30407599 PMC6323923

[B34] Burgers P. M. J. Kunkel T. A. (2017). Eukaryotic DNA Replication Fork. *Annu. Rev. Biochem.* 86 417–438. 10.1146/annurev-biochem-061516-044709 28301743 PMC5597965

[B35] Burrell R. A. Mcclelland S. E. Endesfelder D. Groth P. Weller M. C. Shaikh N. (2013). Replication stress links structural and numerical cancer chromosomal instability. *Nature* 494 492–496. 10.1038/nature11935 23446422 PMC4636055

[B36] Byun T. S. Pacek M. Yee M.-C. Walter J. C. Cimprich K. A. (2005). Functional uncoupling of MCM helicase and DNA polymerase activities activates the ATR-dependent checkpoint. *Genes Dev.* 19 1040–1052. 10.1101/gad.1301205 15833913 PMC1091739

[B37] Cang Y. Zhang J. Nicholas S. A. Bastien J. Li B. Zhou P. (2006). Deletion of DDB1 in mouse brain and lens leads to p53-dependent elimination of proliferating cells. *Cell* 127 929–940. 10.1016/j.cell.2006.09.045 17129780

[B38] Carver E. A. Jiang R. Lan Y. Oram K. F. Gridley T. (2001). The mouse snail gene encodes a key regulator of the epithelial-mesenchymal transition. *Mol. Cell. Biol.* 21 8184–8188. 10.1128/MCB.21.23.8184-8188.2001 11689706 PMC99982

[B39] Celeste A. Petersen S. Romanienko P. J. Fernandez-Capetillo O. Chen H. T. Sedelnikova O. A. (2002). Genomic instability in mice lacking histone H2AX. *Science* 296 922–927. 10.1126/science.1069398 11934988 PMC4721576

[B40] Celli G. B. Denchi E. L. De Lange T. (2006). Ku70 stimulates fusion of dysfunctional telomeres yet protects chromosome ends from homologous recombination. *Nat. Cell Biol.* 8 885–890. 10.1038/ncb1444 16845382

[B41] Chang H. H. Y. Pannunzio N. R. Adachi N. Lieber M. R. (2017). Non-homologous DNA end joining and alternative pathways to double-strand break repair. *Nat. Rev. Mol. Cell Biol.* 18 495–506. 10.1038/nrm.2017.48 28512351 PMC7062608

[B42] Chapman J. R. Barral P. Vannier J. B. Borel V. Steger M. Tomas-Loba A. (2013). RIF1 is essential for 53BP1-dependent nonhomologous end joining and suppression of DNA double-strand break resection. *Mol. Cell* 49 858–871. 10.1016/j.molcel.2013.01.002 23333305 PMC3594748

[B43] Chen C. H. Chu P. C. Lee L. Lien H. W. Lin T. L. Fan C. C. (2012). Disruption of murine mp29/Syf2/Ntc31 gene results in embryonic lethality with aberrant checkpoint response. *PLoS One* 7:e33538. 10.1371/journal.pone.0033538 22448250 PMC3308990

[B44] Chen P.-L. Liu F. Cai S. Lin X. Li A. Chen Y. (2005). Inactivation of CtIP leads to early embryonic lethality mediated by G1 restraint and to tumorigenesis by haploid insufficiency. *Mol. Cell. Biol.* 25 3535–3542. 10.1128/MCB.25.9.3535-3542.2005 15831459 PMC1084307

[B45] Chester N. Kuo F. Kozak C. O’hara C. D. Leder P. (1998). Stage-specific apoptosis, developmental delay, and embryonic lethality in mice homozygous for a targeted disruption in the murine Bloom’s syndrome gene. *Genes Dev.* 12 3382–3393. 10.1101/gad.12.21.3382 9808625 PMC317228

[B46] Cho E. A. Prindle M. J. Dressler G. R. (2003). BRCT domain-containing protein PTIP is essential for progression through mitosis. *Mol. Cell. Biol.* 23 1666–1673. 10.1128/mcb.23.5.1666-1673.2003 12588986 PMC151700

[B47] Choi J.-H. Lindsey-Boltz L. A. Kemp M. Mason A. C. Wold M. S. Sancar A. (2010). Reconstitution of RPA-covered single-stranded DNA-activated ATR-Chk1 signaling. *Proc. Natl. Acad. Sci. U.S.A.* 107 13660–13665. 10.1073/pnas.1007856107 20616048 PMC2922256

[B48] Condic M. L. (2014). Totipotency: what it is and what it is not. *Stem Cells Dev.* 23 796–812. 10.1089/scd.2013.0364 24368070 PMC3991987

[B49] Conklin J. F. Baker J. Sage J. (2012). The RB family is required for the self-renewal and survival of human embryonic stem cells. *Nat. Commun.* 3:1244. 10.1038/ncomms2254 23212373

[B50] Cortez D. (2019). Replication-Coupled DNA Repair. *Mol. Cell* 74 866–876. 10.1016/j.molcel.2019.04.027 31173722 PMC6557297

[B51] Cortez D. Guntuku S. Qin J. Elledge S. J. (2001). ATR and ATRIP: partners in checkpoint signaling. *Science* 294 1713–1716. 10.1126/science.1065521 11721054

[B52] Costantino L. Sotiriou S. K. Rantala J. K. Magin S. Mladenov E. Helleday T. (2014). Break-induced replication repair of damaged forks induces genomic duplications in human cells. *Science* 343 88–91. 10.1126/science.1243211 24310611 PMC4047655

[B53] Couch F. B. Bansbach C. E. Driscoll R. Luzwick J. W. Glick G. G. Betous R. (2013). ATR phosphorylates SMARCAL1 to prevent replication fork collapse. *Genes Dev.* 27 1610–1623. 10.1101/gad.214080.113 23873943 PMC3731549

[B54] Courtot L. Hoffmann J. S. Bergoglio V. (2018). The protective role of dormant origins in response to replicative stress. *Int. J. Mol. Sci.* 19:E3569. 10.3390/ijms19113569 30424570 PMC6274952

[B55] Crossley M. P. Bocek M. Cimprich K. A. (2019). R-Loops as Cellular Regulators and Genomic Threats. *Mol. Cell* 73 398–411. 10.1016/j.molcel.2019.01.024 30735654 PMC6402819

[B56] Davies S. L. North P. S. Hickson I. D. (2007). Role for BLM in replication-fork restart and suppression of origin firing after replicative stress. *Nat. Struct. Mol. Biol.* 14 677–679. 10.1038/nsmb1267 17603497

[B57] Daza-Martin M. Starowicz K. Jamshad M. Tye S. Ronson G. E. Mackay H. L. (2019). Isomerization of BRCA1-BARD1 promotes replication fork protection. *Nature* 571 521–527. 10.1038/s41586-019-1363-4 31270457

[B58] de Boer J. Donker I. De Wit J. Hoeijmakers J. H. Weeda G. (1998). Disruption of the mouse xeroderma pigmentosum group D DNA repair/basal transcription gene results in preimplantation lethality. *Cancer Res.* 58 89–94. 9426063

[B59] de Klein A. Muijtjens M. Van Os R. Verhoeven Y. Smit B. Carr A. M. (2000). Targeted disruption of the cell-cycle checkpoint gene ATR leads to early embryonic lethality in mice. *Curr. Biol.* 10 479–482. 10.1016/s0960-9822(00)00447-4 10801416

[B60] Delacroix S. Wagner J. M. Kobayashi M. Yamamoto K. Karnitz L. M. (2007). The Rad9-Hus1-Rad1 (9-1-1) clamp activates checkpoint signaling via TopBP1. *Genes Dev.* 21 1472–1477. 10.1101/gad.1547007 17575048 PMC1891424

[B61] Denchi E. L. de Lange T. (2007). Protection of telomeres through independent control of ATM and ATR by TRF2 and POT1. *Nature* 448 1068–1071. 10.1038/nature06065 17687332

[B62] Dick F. A. Rubin S. M. (2013). Molecular mechanisms underlying RB protein function. *Nat. Rev. Mol. Cell Biol.* 14 297–306. 10.1038/nrm3567 23594950 PMC4754300

[B63] Dickinson M. E. Flenniken A. M. Ji X. Teboul L. Wong M. D. White J. K. (2016). High-throughput discovery of novel developmental phenotypes. *Nature* 537 508–514. 10.1038/nature19356 27626380 PMC5295821

[B64] Ding X. Ray Chaudhuri A. Callen E. Pang Y. Biswas K. Klarmann K. D. (2016). Synthetic viability by BRCA2 and PARP1/ARTD1 deficiencies. *Nat. Commun.* 7:12425. 10.1038/ncomms12425 27498558 PMC4979061

[B65] Diril M. K. Ratnacaram C. K. Padmakumar V. C. Du T. Wasser M. Coppola V. (2012). Cyclin-dependent kinase 1 (Cdk1) is essential for cell division and suppression of DNA re-replication but not for liver regeneration. *Proc. Natl. Acad. Sci. U S.A.* 109 3826–3831. 10.1073/pnas.1115201109 22355113 PMC3309725

[B66] Dupre A. Boyer-Chatenet L. Gautier J. (2006). Two-step activation of ATM by DNA and the Mre11-Rad50-Nbs1 complex. *Nat. Struct. Mol. Biol.* 13 451–457. 10.1038/nsmb1090 16622404

[B67] Egly J. M. (2001). The 14th Datta Lecture. TFIIH: from transcription to clinic. *FEBS Lett.* 498 124–128. 10.1016/s0014-5793(01)02458-9 11412842

[B68] Feng W. Jasin M. (2017). BRCA2 suppresses replication stress-induced mitotic and G1 abnormalities through homologous recombination. *Nat. Commun.* 8:525. 10.1038/s41467-017-00634-0 28904335 PMC5597640

[B69] Flach J. Bakker S. T. Mohrin M. Conroy P. C. Pietras E. M. Reynaud D. (2014). Replication stress is a potent driver of functional decline in ageing haematopoietic stem cells. *Nature* 512 198–202. 10.1038/nature13619 25079315 PMC4456040

[B70] Fortschegger K. Wagner B. Voglauer R. Katinger H. Sibilia M. Grillari J. (2007). Early embryonic lethality of mice lacking the essential protein SNEV. *Mol. Cell. Biol.* 27 3123–3130. 10.1128/MCB.01188-06 17283042 PMC1899945

[B71] Foster C. T. Dovey O. M. Lezina L. Luo J. L. Gant T. W. Barlev N. (2010). Lysine-specific demethylase 1 regulates the embryonic transcriptome and CoREST stability. *Mol. Cell. Biol.* 30 4851–4863. 10.1128/MCB.00521-10 20713442 PMC2950538

[B72] Foti R. Gnan S. Cornacchia D. Dileep V. Bulut-Karslioglu A. Diehl S. (2016). Nuclear Architecture Organized by Rif1 Underpins the Replication-Timing Program. *Mol. Cell* 61 260–273. 10.1016/j.molcel.2015.12.001 26725008 PMC4724237

[B73] Frank K. M. Sekiguchi J. M. Seidl K. J. Swat W. Rathbun G. A. Cheng H. L. (1998). Late embryonic lethality and impaired V(D)J recombination in mice lacking DNA ligase IV. *Nature* 396 173–177. 10.1038/24172 9823897

[B74] Fukushima T. Matsuzawa S. Kress C. L. Bruey J. M. Krajewska M. Lefebvre S. (2007). Ubiquitin-conjugating enzyme Ubc13 is a critical component of TNF receptor-associated factor (TRAF)-mediated inflammatory responses. *Proc. Natl. Acad. Sci. U.S.A.* 104 6371–6376. 10.1073/pnas.0700548104 17404240 PMC1851032

[B75] Ganuza M. Sáiz-Ladera C. Cañamero M. Gómez G. Schneider R. Blasco M. A. (2012). Genetic inactivation of Cdk7 leads to cell cycle arrest and induces premature aging due to adult stem cell exhaustion. *EMBO J.* 31 2498–2510. 10.1038/emboj.2012.94 22505032 PMC3365431

[B76] Garzon J. Rodriguez R. Kong Z. Chabes A. Rodriguez-Acebes S. Mendez J. (2017). Shortage of dNTPs underlies altered replication dynamics and DNA breakage in the absence of the APC/C cofactor Cdh1. *Oncogene* 36 5808–5818. 10.1038/onc.2017.186 28604743

[B77] Gavet O. Pines J. (2010). Progressive activation of CyclinB1-Cdk1 coordinates entry to mitosis. *Dev. Cell* 18 533–543. 10.1016/j.devcel.2010.02.013 20412769 PMC3325599

[B78] Ge X. Q. Han J. Cheng E. C. Yamaguchi S. Shima N. Thomas J. L. (2015). Embryonic Stem Cells License a High Level of Dormant Origins to Protect the Genome against Replication Stress. *Stem Cell Rep.* 5 185–194. 10.1016/j.stemcr.2015.06.002 26190528 PMC4618655

[B79] Georgakilas A. G. Martin O. A. Bonner W. M. (2017). p21: a two-faced genome guardian. *Trends Mol. Med.* 23 310–319. 10.1016/j.molmed.2017.02.001 28279624

[B80] Ghezraoui H. Piganeau M. Renouf B. Renaud J.-B. Sallmyr A. Ruis B. (2014). Chromosomal translocations in human cells are generated by canonical nonhomologous end-joining. *Mol. Cell* 55 829–842. 10.1016/j.molcel.2014.08.002 25201414 PMC4398060

[B81] Gotter A. L. Manganaro T. Weaver D. R. Kolakowski LF Jr Possidente B. Sriram S. (2000). A time-less function for mouse timeless. *Nat. Neurosci.* 3 755–756. 10903565 10.1038/77653

[B82] Gottlieb T. M. Jackson S. P. (1993). The DNA-dependent protein kinase: requirement for DNA ends and association with Ku antigen. *Cell* 72 131–142. 10.1016/0092-8674(93)90057-w 8422676

[B83] Gu Y. Seidl K. J. Rathbun G. A. Zhu C. Manis J. P. Van Der Stoep N. (1997). Growth retardation and leaky SCID phenotype of Ku70-deficient mice. *Immunity* 7 653–665. 10.1016/s1074-7613(00)80386-6 9390689

[B84] Haahr P. Hoffmann S. Tollenaere M. A. Ho T. Toledo L. I. Mann M. (2016). Activation of the ATR kinase by the RPA-binding protein ETAA1. *Nat. Cell Biol.* 18 1196–1207. 10.1038/ncb3422 27723717

[B85] Hakem R. De La Pompa J. L. Sirard C. Mo R. Woo M. Hakem A. (1996). The tumor suppressor gene Brca1 is required for embryonic cellular proliferation in the mouse. *Cell* 85 1009–1023. 10.1016/s0092-8674(00)81302-1 8674108

[B86] Hamatani T. Carter M. G. Sharov A. A. Ko M. S. H. (2004). Dynamics of global gene expression changes during mouse preimplantation development. *Dev. Cell* 6 117–131. 10.1016/s1534-5807(03)00373-3 14723852

[B87] Harvey S. L. Charlet A. Haas W. Gygi S. P. Kellogg D. R. (2005). Cdk1-dependent regulation of the mitotic inhibitor Wee1. *Cell* 122 407–420. 10.1016/j.cell.2005.05.029 16096060

[B88] Haupt Y. Maya R. Kazaz A. Oren M. (1997). Mdm2 promotes the rapid degradation of p53. *Nature* 387 296–299. 10.1038/387296a0 9153395

[B89] Hemberger M. Nozaki T. Winterhager E. Yamamoto H. Nakagama H. Kamada N. (2003). Parp1-deficiency induces differentiation of ES cells into trophoblast derivatives. *Dev. Biol.* 257 371–381. 10.1016/s0012-1606(03)00097-6 12729565

[B90] Heyer B. S. Macauley A. Behrendtsen O. Werb Z. (2000). Hypersensitivity to DNA damage leads to increased apoptosis during early mouse development. *Genes Dev.* 14 2072–2084. 10950870 PMC316856

[B91] Hiller B. Achleitner M. Glage S. Naumann R. Behrendt R. Roers A. (2012). Mammalian RNase H2 removes ribonucleotides from DNA to maintain genome integrity. *J. Exp. Med.* 209 1419–1426. 10.1084/jem.20120876 22802351 PMC3409502

[B92] Hirschi A. Cecchini M. Steinhardt R. C. Schamber M. R. Dick F. A. Rubin S. M. (2010). An overlapping kinase and phosphatase docking site regulates activity of the retinoblastoma protein. *Nat. Struct. Mol. Biol.* 17 1051–1057. 10.1038/nsmb.1868 20694007 PMC2933323

[B93] Hnizda A. Blundell T. L. (2019). Multicomponent assemblies in DNA-double-strand break repair by NHEJ. *Curr. Opin. Struct. Biol.* 55 154–160. 10.1016/j.sbi.2019.03.026 31125797

[B94] Hockemeyer D. Daniels J.-P. Takai H. De Lange T. (2006). Recent expansion of the telomeric complex in rodents: Two distinct POT1 proteins protect mouse telomeres. *Cell* 126 63–77. 10.1016/j.cell.2006.04.044 16839877

[B95] Hockemeyer D. Sfeir A. J. Shay J. W. Wright W. E. De Lange T. (2005). POT1 protects telomeres from a transient DNA damage response and determines how human chromosomes end. *EMBO J.* 24 2667–2678. 10.1038/sj.emboj.7600733 15973431 PMC1176460

[B96] Hoki Y. Araki R. Fujimori A. Ohhata T. Koseki H. Fukumura R. (2003). Growth retardation and skin abnormalities of the Recql4-deficient mouse. *Hum. Mol. Genet.* 12 2293–2299. 10.1093/hmg/ddg254 12915449

[B97] Horii T. Yamamoto M. Morita S. Kimura M. Nagao Y. Hatada I. (2015). p53 suppresses tetraploid development in mice. *Sci. Rep.* 5:8907. 10.1038/srep08907 25752699 PMC4354145

[B98] Humięcka M. Szpila M. Kłoś P. Maleszewski M. Szczepańska K. (2017). Mouse blastomeres acquire ability to divide asymmetrically before compaction. *PLoS One* 12:e0175032. 10.1371/journal.pone.0175032 28362853 PMC5376319

[B99] Hustedt N. Durocher D. (2016). The control of DNA repair by the cell cycle. *Nat. Cell Biol.* 19 1–9. 10.1038/ncb3452 28008184

[B100] Jackson S. P. Durocher D. (2013). Regulation of DNA damage responses by ubiquitin and SUMO. *Mol. Cell* 49 795–807. 10.1016/j.molcel.2013.01.017 23416108

[B101] Jacome A. Gutierrez-Martinez P. Schiavoni F. Tenaglia E. Martinez P. Rodriguez-Acebes S. (2015). NSMCE2 suppresses cancer and aging in mice independently of its SUMO ligase activity. *EMBO J.* 34 2604–2619. 10.15252/embj.201591829 26443207 PMC4641528

[B102] Jain A. K. Xi Y. Mccarthy R. Allton K. Akdemir K. C. Patel L. R. (2016). LncPRESS1 is a p53-Regulated LncRNA that safeguards pluripotency by disrupting SIRT6-mediated de-acetylation of histone H3K56. *Mol. Cell* 64 967–981. 10.1016/j.molcel.2016.10.039 27912097 PMC5137794

[B103] Jeon Y. Ko E. Lee K. Y. Ko M. J. Park S. Y. Kang J. (2011). TopBP1 deficiency causes an early embryonic lethality and induces cellular senescence in primary cells. *J. Biol. Chem.* 286 5414–5422. 10.1074/jbc.M110.189704 21149450 PMC3037654

[B104] Jones S. N. Roe A. E. Donehower L. A. Bradley A. (1995). Rescue of embryonic lethality in Mdm2-deficient mice by absence of p53. *Nature* 378 206–208. 10.1038/378206a0 7477327

[B105] Jukam D. Shariati S. A. M. Skotheim J. M. (2017). Zygotic genome activation in vertebrates. *Dev. Cell* 42 316–332. 28829942 10.1016/j.devcel.2017.07.026 PMC5714289

[B106] Kafer G. R. Li X. Horii T. Suetake I. Tajima S. Hatada I. (2016). 5-Hydroxymethylcytosine Marks Sites of DNA Damage and Promotes Genome Stability. *Cell Rep.* 14 1283–1292. 10.1016/j.celrep.2016.01.035 26854228

[B107] Kemp M. G. Akan Z. Yilmaz S. Grillo M. Smith-Roe S. L. Kang T. H. (2010). Tipin-replication protein A interaction mediates Chk1 phosphorylation by ATR in response to genotoxic stress. *J. Biol. Chem.* 285 16562–16571. 10.1074/jbc.M110.110304 20233725 PMC2878033

[B108] Kent L. N. Leone G. (2019). The broken cycle: E2F dysfunction in cancer. *Nat. Rev. Cancer* 19 326–338. 10.1038/s41568-019-0143-7 31053804

[B109] Kent T. Chandramouly G. Mcdevitt S. M. Ozdemir A. Y. Pomerantz R. T. (2015). Mechanism of microhomology-mediated end-joining promoted by human DNA polymerase θ. *Nat. Struct. Mol. Biol.* 22 230–237. 10.1038/nsmb.2961 25643323 PMC4351179

[B110] Kim J. M. Nakao K. Nakamura K. Saito I. Katsuki M. Arai K.-I. (2002). Inactivation of Cdc7 kinase in mouse ES cells results in S-phase arrest and p53-dependent cell death. *EMBO J.* 21 2168–2179. 10.1093/emboj/21.9.2168 11980714 PMC125997

[B111] Kon N. Kobayashi Y. Li M. Brooks C. L. Ludwig T. Gu W. (2010). Inactivation of HAUSP in vivo modulates p53 function. *Oncogene* 29 1270–1279. 10.1038/onc.2009.427 19946331 PMC2857765

[B112] Kramara J. Osia B. Malkova A. (2018). Break-induced replication: the Where. The Why, and The How. *Trends Genet.* 34 518–531. 10.1016/j.tig.2018.04.002 29735283 PMC6469874

[B113] Kumagai A. Lee J. Yoo H. Y. Dunphy W. G. (2006). TopBP1 activates the ATR-ATRIP complex. *Cell* 124 943–955. 16530042 10.1016/j.cell.2005.12.041

[B114] Kurimasa A. Ouyang H. Dong L. J. Wang S. Li X. Cordon-Cardo C. (1999). Catalytic subunit of DNA-dependent protein kinase: impact on lymphocyte development and tumorigenesis. *Proc. Natl. Acad. Sci. U.S.A.* 96 1403–1408. 10.1073/pnas.96.4.1403 9990036 PMC15475

[B115] Kuznetsov S. G. Haines D. C. Martin B. K. Sharan S. K. (2009). Loss of Rad51c leads to embryonic lethality and modulation of Trp53-dependent tumorigenesis in mice. *Cancer Res.* 69 863–872. 10.1158/0008-5472.CAN-08-3057 19155299 PMC2754281

[B116] Lamm N. Ben-David U. Golan-Lev T. Storchova Z. Benvenisty N. Kerem B. (2016). Genomic instability in human pluripotent stem cells arises from replicative stress and chromosome condensation defects. *Cell Stem Cell* 18 253–261. 10.1016/j.stem.2015.11.003 26669899

[B117] Larsen E. Gran C. Saether B. E. Seeberg E. Klungland A. (2003). Proliferation failure and gamma radiation sensitivity of Fen1 null mutant mice at the blastocyst stage. *Mol. Cell. Biol.* 23 5346–5353. 10.1128/mcb.23.15.5346-5353.2003 12861020 PMC165721

[B118] Laurent A. Blasi F. (2015). Differential DNA damage signalling and apoptotic threshold correlate with mouse epiblast-specific hypersensitivity to radiation. *Development* 142 3675–3685. 10.1242/dev.125708 26395482

[B119] Lecona E. Rodriguez-Acebes S. Specks J. Lopez-Contreras A. J. Ruppen I. Murga M. (2016). USP7 is a SUMO deubiquitinase essential for DNA replication. *Nat. Struct. Mol. Biol.* 23 270–277. 10.1038/nsmb.3185 26950370 PMC4869841

[B120] Lee J. H. Paull T. T. (2005). ATM activation by DNA double-strand breaks through the Mre11-Rad50-Nbs1 complex. *Science* 308 551–554. 10.1126/science.1108297 15790808

[B121] Lee S. Kim J. Y. Kim Y. J. Seok K. O. Kim J. H. Chang Y. J. (2012). Nucleolar protein GLTSCR2 stabilizes p53 in response to ribosomal stresses. *Cell Death Differ.* 19 1613–1622. 10.1038/cdd.2012.40 22522597 PMC3438492

[B122] Lemaçon D. Jackson J. Quinet A. Brickner J. R. Li S. Yazinski S. (2017). MRE11 and EXO1 nucleases degrade reversed forks and elicit MUS81-dependent fork rescue in BRCA2-deficient cells. *Nat. Commun.* 8:860. 10.1038/s41467-017-01180-5 29038425 PMC5643552

[B123] Li A. Blow J. J. (2005). Cdt1 downregulation by proteolysis and geminin inhibition prevents DNA re-replication in Xenopus. *EMBO J.* 24 395–404. 10.1038/sj.emboj.7600520 15616577 PMC545810

[B124] Li B. Ruiz J. C. Chun K. T. (2002). CUL-4A is critical for early embryonic development. *Mol. Cell. Biol.* 22 4997–5005. 10.1128/mcb.22.14.4997-5005.2002 12077329 PMC139768

[B125] Li G. Alt F. W. Cheng H. L. Brush J. W. Goff P. H. Murphy M. M. (2008). Lymphocyte-specific compensation for XLF/cernunnos end-joining functions in V(D)J recombination. *Mol. Cell* 31 631–640. 10.1016/j.molcel.2008.07.017 18775323 PMC2630261

[B126] Li M. He Y. Dubois W. Wu X. Shi J. Huang J. (2012). Distinct regulatory mechanisms and functions for p53-activated and p53-repressed DNA damage response genes in embryonic stem cells. *Mol. Cell* 46 30–42. 10.1016/j.molcel.2012.01.020 22387025 PMC3327774

[B127] Lim D. S. Hasty P. (1996). A mutation in mouse rad51 results in an early embryonic lethal that is suppressed by a mutation in p53. *Mol. Cell. Biol.* 16 7133–7143. 10.1128/mcb.16.12.7133 8943369 PMC231717

[B128] Limas J. C. Cook J. G. (2019). Preparation for DNA replication: the key to a successful S phase. *FEBS Lett.* 593 2853–2867. 10.1002/1873-3468.13619 31556113 PMC6817399

[B129] Lin T. Chao C. Saito S. Mazur S. J. Murphy M. E. Appella E. (2005). p53 induces differentiation of mouse embryonic stem cells by suppressing Nanog expression. *Nat. Cell Biol.* 7 165–171. 10.1038/ncb1211 15619621

[B130] Lin W. Sampathi S. Dai H. Liu C. Zhou M. Hu J. (2013). Mammalian DNA2 helicase/nuclease cleaves G-quadruplex DNA and is required for telomere integrity. *EMBO J.* 32 1425–1439. 10.1038/emboj.2013.88 23604072 PMC3655473

[B131] Liu C. Vyas A. Kassab M. A. Singh A. K. Yu X. (2017). The role of poly ADP-ribosylation in the first wave of DNA damage response. *Nucleic Acids Res.* 45 8129–8141. 10.1093/nar/gkx565 28854736 PMC5737498

[B132] Liu C.-L. Yu I. S. Pan H.-W. Lin S.-W. Hsu H.-C. (2007). L2dtl is essential for cell survival and nuclear division in early mouse embryonic development. *J. Biol. Chem.* 282 1109–1118. 10.1074/jbc.M606535200 17107960

[B133] Liu D. Keijzers G. Rasmussen L. J. (2017). DNA mismatch repair and its many roles in eukaryotic cells. *Mutat. Res.* 773 174–187. 10.1016/j.mrrev.2017.07.001 28927527

[B134] Liu Q. Guntuku S. Cui X. S. Matsuoka S. Cortez D. Tamai K. (2000). Chk1 is an essential kinase that is regulated by Atr and required for the G(2)/M DNA damage checkpoint. *Genes Dev.* 14 1448–1459. 10859164 PMC316686

[B135] Lou J. Scipioni L. Wright B. K. Bartolec T. K. Zhang J. Masamsetti V. P. (2019). Phasor histone FLIM-FRET microscopy quantifies spatiotemporal rearrangement of chromatin architecture during the DNA damage response. *Proc. Natl. Acad. Sci. U.S.A.* 116 7323–7332. 10.1073/pnas.1814965116 30918123 PMC6462080

[B136] Lou Z. Minter-Dykhouse K. Franco S. Gostissa M. Rivera M. A. Celeste A. (2006). MDC1 maintains genomic stability by participating in the amplification of ATM-dependent DNA damage signals. *Mol. Cell* 21 187–200. 10.1016/j.molcel.2005.11.025 16427009

[B137] Lu L. Y. Wood J. L. Minter-Dykhouse K. Ye L. Saunders T. L. Yu X. (2008). Polo-like kinase 1 is essential for early embryonic development and tumor suppression. *Mol. Cell. Biol.* 28 6870–6876. 10.1128/MCB.00392-08 18794363 PMC2573299

[B138] Ludwig T. Chapman D. L. Papaioannou V. E. Efstratiadis A. (1997). Targeted mutations of breast cancer susceptibility gene homologs in mice: lethal phenotypes of Brca1, Brca2, Brca1/Brca2, Brca1/p53, and Brca2/p53 nullizygous embryos. *Genes Dev.* 11 1226–1241. 10.1101/gad.11.10.1226 9171368

[B139] Lukas C. Savic V. Bekker-Jensen S. Doil C. Neumann B. Pedersen R. S. (2011). 53BP1 nuclear bodies form around DNA lesions generated by mitotic transmission of chromosomes under replication stress. *Nat. Cell Biol.* 13 243–253. 10.1038/ncb2201 21317883

[B140] Luo G. Yao M. S. Bender C. F. Mills M. Bladl A. R. Bradley A. (1999). Disruption of mRad50 causes embryonic stem cell lethality, abnormal embryonic development, and sensitivity to ionizing radiation. *Proc. Natl. Acad. Sci. U.S.A.* 96 7376–7381. 10.1073/pnas.96.13.7376 10377422 PMC22093

[B141] Luo L. Z. Gopalakrishna-Pillai S. Nay S. L. Park S. W. Bates S. E. Zeng X. (2012). DNA repair in human pluripotent stem cells is distinct from that in non-pluripotent human cells. *PLoS One* 7:e30541. 10.1371/journal.pone.0030541 22412831 PMC3295811

[B142] Ly P. Cleveland D. W. (2017). Rebuilding chromosomes after catastrophe: emerging mechanisms of chromothripsis. *Trends Cell Biol.* 27 917–930. 10.1016/j.tcb.2017.08.005 28899600 PMC5696049

[B143] Machwe A. Karale R. Xu X. Liu Y. Orren D. K. (2011). The Werner and Bloom syndrome proteins help resolve replication blockage by converting (regressed) holliday junctions to functional replication forks. *Biochemistry* 50 6774–6788. 10.1021/bi2001054 21736299 PMC3153593

[B144] Maciejowski J. Li Y. Bosco N. Campbell P. J. De Lange T. (2015). Chromothripsis and Kataegis Induced by Telomere Crisis. *Cell* 163 1641–1654. 10.1016/j.cell.2015.11.054 26687355 PMC4687025

[B145] Mailand N. Gibbs-Seymour I. Bekker-Jensen S. (2013). Regulation of PCNA-protein interactions for genome stability. *Nat. Rev. Mol. Cell Biol.* 14 269–282. 10.1038/nrm3562 23594953

[B146] Mancini A. Niemann-Seyde S. C. Pankow R. El Bounkari O. Klebba-Farber S. Koch A. (2010). THOC5/FMIP, an mRNA export TREX complex protein, is essential for hematopoietic primitive cell survival in vivo. *BMC Biol* 8:1. 10.1186/1741-7007-8-1 20051105 PMC2806247

[B147] Mandal R. Strebhardt K. (2013). Plk1: unexpected roles in DNA replication. *Cell Res.* 23 1251–1253. 10.1038/cr.2013.130 24042259 PMC3817549

[B148] Mankouri H. W. Huttner D. Hickson I. D. (2013). How unfinished business from S-phase affects mitosis and beyond. *EMBO J.* 32 2661–2671. 10.1038/emboj.2013.211 24065128 PMC3801442

[B149] Maréchal A. Li J.-M. Ji X. Y. Wu C.-S. Yazinski S. A. Nguyen H. D. (2014). PRP19 transforms into a sensor of RPA-ssDNA after DNA damage and drives ATR activation via a ubiquitin-mediated circuitry. *Mol. Cell* 53 235–246. 10.1016/j.molcel.2013.11.002 24332808 PMC3946837

[B150] Maréchal A. Zou L. (2013). DNA damage sensing by the ATM and ATR kinases. *Cold Spring Harb. Perspect. Biol.* 5:a012716. 10.1101/cshperspect.a012716 24003211 PMC3753707

[B151] Masamsetti V. P. Low R. R. J. Mak K. S. O’connor A. Riffkin C. D. Lamm N. (2019). Replication stress induces mitotic death through parallel pathways regulated by WAPL and telomere deprotection. *Nat. Commun.* 10:4224. 10.1038/s41467-019-12255-w 31530811 PMC6748914

[B152] Masuda K. Ouchida R. Takeuchi A. Saito T. Koseki H. Kawamura K. (2005). DNA polymerase theta contributes to the generation of C/G mutations during somatic hypermutation of Ig genes. *Proc. Natl. Acad. Sci. U.S.A.* 102 13986–13991. 10.1073/pnas.0505636102 16172387 PMC1236561

[B153] Mateos-Gomez P. A. Gong F. Nair N. Miller K. M. Lazzerini-Denchi E. Sfeir A. (2015). Mammalian polymerase θ promotes alternative NHEJ and suppresses recombination. *Nature* 518 254–257. 10.1038/nature14157 25642960 PMC4718306

[B154] Mateos-Gomez P. A. Kent T. Deng S. K. Mcdevitt S. Kashkina E. Hoang T. M. (2017). The helicase domain of Polθ counteracts RPA to promote alt-NHEJ. *Nat. Struct. Mol. Biol.* 24 1116–1123. 10.1038/nsmb.3494 29058711 PMC6047744

[B155] Matson J. P. Dumitru R. Coryell P. Baxley R. M. Chen W. Twaroski K. (2017). Rapid DNA replication origin licensing protects stem cell pluripotency. *eLife* 6:e30473. 10.7554/eLife.30473 PMC5720591 29148972

[B156] Maynard S. Swistowska A. M. Lee J. W. Liu Y. Liu S. T. Da Cruz A. B. (2008). Human embryonic stem cells have enhanced repair of multiple forms of DNA damage. *Stem Cells* 26 2266–2274. 10.1634/stemcells.2007-1041 18566332 PMC2574957

[B157] McCarthy E. E. Celebi J. T. Baer R. Ludwig T. (2003). Loss of Bard1, the heterodimeric partner of the Brca1 tumor suppressor, results in early embryonic lethality and chromosomal instability. *Mol. Cell. Biol.* 23 5056–5063. 10.1128/mcb.23.14.5056-5063.2003 12832489 PMC162231

[B158] Mello S. S. Attardi L. D. (2018). Deciphering p53 signaling in tumor suppression. *Curr. Opin. Cell Biol.* 51 65–72. 10.1016/j.ceb.2017.11.005 29195118 PMC5949255

[B159] Meryet-Figuiere M. Alaei-Mahabadi B. Ali M. M. Mitra S. Subhash S. Pandey G. K. (2014). Temporal separation of replication and transcription during S-phase progression. *Cell Cycle* 13 3241–3248. 10.4161/15384101.2014.953876 25485504 PMC4615114

[B160] Mijic S. Zellweger R. Chappidi N. Berti M. Jacobs K. Mutreja K. (2017). Replication fork reversal triggers fork degradation in BRCA2-defective cells. *Nat. Commun.* 8:859. 10.1038/s41467-017-01164-5 29038466 PMC5643541

[B161] Miller T. C. R. Locke J. Greiwe J. F. Diffley J. F. X. Costa A. (2019). Mechanism of head-to-head MCM double-hexamer formation revealed by cryo-EM. *Nature* 575 704–710. 10.1038/s41586-019-1768-0 31748745 PMC6887548

[B162] Min J.-N. Tian Y. Xiao Y. Wu L. Li L. Chang S. (2013). The mINO80 chromatin remodeling complex is required for efficient telomere replication and maintenance of genome stability. *Cell Res.* 23 1396–1413. 10.1038/cr.2013.113 23979016 PMC3847565

[B163] Minocherhomji S. Ying S. Bjerregaard V. A. Bursomanno S. Aleliunaite A. Wu W. (2015). Replication stress activates DNA repair synthesis in mitosis. *Nature* 528 286–290. 10.1038/nature16139 26633632

[B164] Mohammed H. Hernando-Herraez I. Savino A. Scialdone A. Macaulay I. Mulas C. (2017). Single-cell landscape of transcriptional heterogeneity and cell fate decisions during mouse early gastrulation. *Cell Rep.* 20 1215–1228. 10.1016/j.celrep.2017.07.009 28768204 PMC5554778

[B165] Mohri T. Ueno M. Nagahama Y. Gong Z.-Y. Asano M. Oshima H. (2013). Requirement of SLD5 for early embryogenesis. *PLoS One* 8:e78961. 10.1371/journal.pone.0078961 24244394 PMC3823970

[B166] Morris S. A. Teo R. T. Li H. Robson P. Glover D. M. Zernicka-Goetz M. (2010). Origin and formation of the first two distinct cell types of the inner cell mass in the mouse embryo. *Proc. Natl. Acad. Sci. U.S.A.* 107 6364–6369. 10.1073/pnas.0915063107 20308546 PMC2852013

[B167] Murga M. Bunting S. Montaña M. F. Soria R. Mulero F. Cañamero M. (2009). A mouse model of ATR-Seckel shows embryonic replicative stress and accelerated aging. *Nat. Genet.* 41 891–898. 10.1038/ng.420 19620979 PMC2902278

[B168] Murray S. A. Morgan J. L. Kane C. Sharma Y. Heffner C. S. Lake J. (2010). Mouse gestation length is genetically determined. *PLoS One* 5:e12418. 10.1371/journal.pone.0012418 20811634 PMC2928290

[B169] Myers K. Gagou M. E. Zuazua-Villar P. Rodriguez R. Meuth M. (2009). ATR and Chk1 suppress a caspase-3-dependent apoptotic response following DNA replication stress. *PLoS Genet.* 5:e1000324. 10.1371/journal.pgen.1000324 19119425 PMC2607051

[B170] Navadgi-Patil V. M. Burgers P. M. (2009). A tale of two tails: activation of DNA damage checkpoint kinase Mec1/ATR by the 9-1-1 clamp and by Dpb11/TopBP1. *DNA Repair* 8 996–1003. 10.1016/j.dnarep.2009.03.011 19464966 PMC2725207

[B171] Neelsen K. J. Lopes M. (2015). Replication fork reversal in eukaryotes: from dead end to dynamic response. *Nat. Rev. Mol. Cell Biol.* 16 207–220. 10.1038/nrm3935 25714681

[B172] Nigg E. A. (2001). Mitotic kinases as regulators of cell division and its checkpoints. *Nat. Rev. Mol. Cell Biol.* 2 21–32. 10.1038/35048096 11413462

[B173] Nishitani H. Taraviras S. Lygerou Z. Nishimoto T. (2001). The human licensing factor for DNA replication Cdt1 accumulates in G1 and is destabilized after initiation of S-phase. *J. Biol. Chem.* 276 44905–44911. 10.1074/jbc.M105406200 11555648

[B174] Nozaki T. Masutani M. Watanabe M. Ochiya T. Hasegawa F. Nakagama H. (1999). Syncytiotrophoblastic giant cells in teratocarcinoma-like tumors derived from Parp-disrupted mouse embryonic stem cells. *Proc. Natl. Acad. Sci. U.S.A.* 96 13345–13350. 10.1073/pnas.96.23.13345 10557323 PMC23950

[B175] Nussenzweig A. Chen C. Da Costa Soares V. Sanchez M. Sokol K. Nussenzweig M. C. (1996). Requirement for Ku80 in growth and immunoglobulin V(D)J recombination. *Nature* 382 551–555. 10.1038/382551a0 8700231

[B176] Ochi T. Blackford A. N. Coates J. Jhujh S. Mehmood S. Tamura N. (2015). DNA repair. *PAXX, a paralog of XRCC*4 and XLF, interacts with Ku to promote DNA double-strand break repair. *Science* 347 185–188. 10.1126/science.1261971 25574025 PMC4338599

[B177] Ochs F. Karemore G. Miron E. Brown J. Sedlackova H. Rask M. B. (2019). Stabilization of chromatin topology safeguards genome integrity. *Nature* 574 571–574. 10.1038/s41586-019-1659-4 31645724

[B178] O’Connell M. J. Raleigh J. M. Verkade H. M. Nurse P. (1997). Chk1 is a wee1 kinase in the G2 DNA damage checkpoint inhibiting cdc2 by Y15 phosphorylation. *EMBO J.* 16 545–554. 10.1093/emboj/16.3.545 9034337 PMC1169658

[B179] Orthwein A. Fradet-Turcotte A. Noordermeer S. M. Canny M. D. Brun C. M. Strecker J. (2014). Mitosis inhibits DNA double-strand break repair to guard against telomere fusions. *Science* 344 189–193. 10.1126/science.1248024 24652939

[B180] Ouyang H. Nussenzweig A. Kurimasa A. Soares V. C. Li X. Cordon-Cardo C. (1997). Ku70 is required for DNA repair but not for T cell antigen receptor gene recombination *in vivo*. *J. Exp. Med.* 186 921–929. 10.1084/jem.186.6.921 9294146 PMC2199057

[B181] Paim L. M. G. FitzHarris G. (2019). Tetraploidy causes chromosomal instability in acentriolar mouse embryos. *Nat. Commun.* 10:4834. 10.1038/s41467-019-12772-8 31645568 PMC6811537

[B182] Park E. Kim J. M. Primack B. Weinstock D. M. Moreau L. A. Parmar K. (2013). Inactivation of Uaf1 causes defective homologous recombination and early embryonic lethality in mice. *Mol. Cell. Biol.* 33 4360–4370. 10.1128/MCB.00870-13 24001775 PMC3838189

[B183] Pilzecker B. Buoninfante O. A. Van Den Berk P. Lancini C. Song J. Y. Citterio E. (2017). DNA damage tolerance in hematopoietic stem and progenitor cells in mice. *Proc. Natl. Acad. Sci. U.S.A.* 114 E6875–E6883. 10.1073/pnas.1706508114 28761001 PMC5565453

[B184] Pintard L. Archambault V. (2018). A unified view of spatio-temporal control of mitotic entry: Polo kinase as the key. *Open Biol.* 8:180114. 10.1098/rsob.180114 30135239 PMC6119860

[B185] Pizzi S. Sertic S. Orcesi S. Cereda C. Bianchi M. Jackson A. P. (2015). Reduction of hRNase H2 activity in Aicardi-Goutieres syndrome cells leads to replication stress and genome instability. *Hum. Mol. Genet.* 24 649–658. 10.1093/hmg/ddu485 25274781 PMC4291245

[B186] Poli J. Gasser S. M. Papamichos-Chronakis M. (2017). The INO80 remodeller in transcription, replication and repair. *Philos. Trans. R. Soc. Lond. B Biol. Sci.* 372:20160290. 10.1098/rstb.2016.0290 28847827 PMC5577468

[B187] Pollard P. J. Spencer-Dene B. Shukla D. Howarth K. Nye E. El-Bahrawy M. (2007). Targeted inactivation of fh1 causes proliferative renal cyst development and activation of the hypoxia pathway. *Cancer Cell* 11 311–319. 10.1016/j.ccr.2007.02.005 17418408

[B188] Power M. A. Tam P. P. (1993). Onset of gastrulation, morphogenesis and somitogenesis in mouse embryos displaying compensatory growth. *Anat. Embryol.* 187 493–504. 10.1007/BF00174425 8342794

[B189] Przetocka S. Porro A. Bolck H. A. Walker C. Lezaja A. Trenner A. (2018). CtIP-mediated fork protection synergizes with BRCA1 to suppress genomic instability upon DNA replication stress. *Mol. Cell* 72 10.1016/j.molcel.2018.09.014 30344097

[B190] Puebla-Osorio N. Lacey D. B. Alt F. W. Zhu C. (2006). Early embryonic lethality due to targeted inactivation of DNA ligase III. *Mol. Cell. Biol.* 26 3935–3941. 10.1128/MCB.26.10.3935-3941.2006 16648486 PMC1489003

[B191] Quinet A. Lemaçon D. Vindigni A. (2017). Replication fork reversal: players and guardians. *Mol. Cell* 68 830–833. 10.1016/j.molcel.2017.11.022 29220651 PMC5895179

[B192] Ray D. Terao Y. Nimbalkar D. Hirai H. Osmundson E. C. Zou X. (2007). Hemizygous disruption of Cdc25A inhibits cellular transformation and mammary tumorigenesis in mice. *Cancer Res.* 67 6605–6611. 10.1158/0008-5472.CAN-06-4815 17638870

[B193] Ray Chaudhuri A. Callen E. Ding X. Gogola E. Duarte A. A. Lee J. E. (2016). Replication fork stability confers chemoresistance in BRCA-deficient cells. *Nature* 535 382–387. 10.1038/nature18325 27443740 PMC4959813

[B194] Ray Chaudhuri A. Nussenzweig A. (2017). The multifaceted roles of PARP1 in DNA repair and chromatin remodelling. *Nat. Rev. Mol. Cell Biol.* 18 610–621. 10.1038/nrm.2017.53 28676700 PMC6591728

[B195] Reinhardt H. C. Schumacher B. (2012). The p53 network: cellular and systemic DNA damage responses in aging and cancer. *Trends Genet.* 28 128–136. 10.1016/j.tig.2011.12.002 22265392 PMC4120491

[B196] Roa S. Avdievich E. Peled J. U. Maccarthy T. Werling U. Kuang F. L. (2008). Ubiquitylated PCNA plays a role in somatic hypermutation and class-switch recombination and is required for meiotic progression. *Proc. Natl. Acad. Sci. U.S.A.* 105 16248–16253. 10.1073/pnas.0808182105 18854411 PMC2571010

[B197] Rogakou E. P. Pilch D. R. Orr A. H. Ivanova V. S. Bonner W. M. (1998). DNA double-stranded breaks induce histone H2AX phosphorylation on serine 139. *J. Biol. Chem.* 273 5858–5868. 10.1074/jbc.273.10.5858 9488723

[B198] Roper S. J. Chrysanthou S. Senner C. E. Sienerth A. Gnan S. Murray A. (2014). ADP-ribosyltransferases Parp1 and Parp7 safeguard pluripotency of ES cells. *Nucleic Acids Res.* 42 8914–8927. 10.1093/nar/gku591 25034692 PMC4132717

[B199] Rossi D. J. Londesborough A. Korsisaari N. Pihlak A. Lehtonen E. Henkemeyer M. (2001). Inability to enter S phase and defective RNA polymerase II CTD phosphorylation in mice lacking Mat1. *EMBO J.* 20 2844–2856. 10.1093/emboj/20.11.2844 11387217 PMC125252

[B200] Saldivar J. C. Cortez D. Cimprich K. A. (2017). The essential kinase ATR: ensuring faithful duplication of a challenging genome. *Nat. Rev. Mol. Cell Biol.* 18 622–636. 10.1038/nrm.2017.67 28811666 PMC5796526

[B201] Sasaki M. Kawahara K. Nishio M. Mimori K. Kogo R. Hamada K. (2011). Regulation of the MDM2-P53 pathway and tumor growth by PICT1 via nucleolar RPL11. *Nat. Med.* 17 944–951. 10.1038/nm.2392 21804542 PMC4578312

[B202] Schärer O. D. (2013). Nucleotide excision repair in eukaryotes. *Cold Spring Harb. Perspect. Biol.* 5:a012609. 10.1101/cshperspect.a012609 24086042 PMC3783044

[B203] Schlacher K. Christ N. Siaud N. Egashira A. Wu H. Jasin M. (2011). Double-strand break repair-independent role for BRCA2 in blocking stalled replication fork degradation by MRE11. *Cell* 145 529–542. 10.1016/j.cell.2011.03.041 21565612 PMC3261725

[B204] Scully R. Panday A. Elango R. Willis N. A. (2019). DNA double-strand break repair-pathway choice in somatic mammalian cells. *Nat. Rev. Mol. Cell Biol.* 20 698–714. 10.1038/s41580-019-0152-0 31263220 PMC7315405

[B205] Sfeir A. Symington L. S. (2015). Microhomology-mediated end joining: a back-up survival mechanism or dedicated pathway? *Trends Biochem. Sci.* 40 701–714. 10.1016/j.tibs.2015.08.006 26439531 PMC4638128

[B206] Shakya R. Szabolcs M. Mccarthy E. Ospina E. Basso K. Nandula S. (2008). The basal-like mammary carcinomas induced by Brca1 or Bard1 inactivation implicate the BRCA1/BARD1 heterodimer in tumor suppression. *Proc. Natl. Acad. Sci. U.S.A.* 105 7040–7045. 10.1073/pnas.0711032105 18443292 PMC2365565

[B207] Sharan S. K. Morimatsu M. Albrecht U. Lim D. S. Regel E. Dinh C. (1997). Embryonic lethality and radiation hypersensitivity mediated by Rad51 in mice lacking Brca2. *Nature* 386 804–810. 10.1038/386804a0 9126738

[B208] Shima N. Munroe R. J. Schimenti J. C. (2004). The mouse genomic instability mutation chaos1 is an allele of Polq that exhibits genetic interaction with Atm. *Mol. Cell. Biol.* 24 10381–10389. 10.1128/MCB.24.23.10381-10389.2004 15542845 PMC529050

[B209] Shimada M. Dumitrache L. C. Russell H. R. Mckinnon P. J. (2015). Polynucleotide kinase-phosphatase enables neurogenesis via multiple DNA repair pathways to maintain genome stability. *EMBO J.* 34 2465–2480. 10.15252/embj.201591363 26290337 PMC4601665

[B210] Shu Z. Smith S. Wang L. Rice M. C. Kmiec E. B. (1999). Disruption of muREC2/RAD51L1 in mice results in early embryonic lethality which can Be partially rescued in a p53(-/-) background. *Mol. Cell. Biol.* 19 8686–8693. 10.1128/mcb.19.12.8686 10567591 PMC85012

[B211] Shui J. W. Hu M. C. Tan T. H. (2007). Conditional knockout mice reveal an essential role of protein phosphatase 4 in thymocyte development and pre-T-cell receptor signaling. *Mol. Cell. Biol.* 27 79–91. 10.1128/MCB.00799-06 17060460 PMC1800666

[B212] Smith T. G. Laval S. Chen F. Rock M. J. Strachan T. Peters H. (2014). Neural crest cell-specific inactivation of Nipbl or Mau2 during mouse development results in a late onset of craniofacial defects. *Genesis* 52 687–694. 10.1002/dvg.22780 24700590

[B213] Snow M. H. Tam P. P. (1979). Is compensatory growth a complicating factor in mouse teratology? *Nature* 279 555–557. 10.1038/279555a0 571964

[B214] Snow M. H. L. (1976). “Embryo Growth during the Immediate Postimplantation Period,” in *Embryogenesis in Mammals: Ciba Foundation Symposium 40 - Embryogenesis in Mammals*, eds Elliott K. O’Connor M. (Amsterdam: Elsevier), 53–70.

[B215] Snow M. H. L. (1977). Gastrulation in the mouse: growth and regionalization of the epiblast. *Development* 42 293–303. 10.1111/dgd.12568 30368783

[B216] Sobinoff A. P. Allen J. A. Neumann A. A. Yang S. F. Walsh M. E. Henson J. D. (2017). BLM and SLX4 play opposing roles in recombination-dependent replication at human telomeres. *EMBO J.* 36 2907–2919. 10.15252/embj.201796889 28877996 PMC5623873

[B217] Somyajit K. Saxena S. Babu S. Mishra A. Nagaraju G. (2015). Mammalian RAD51 paralogs protect nascent DNA at stalled forks and mediate replication restart. *Nucleic Acids Res.* 43 9835–9855. 10.1093/nar/gkv880 26354865 PMC4787763

[B218] Sotiriou S. K. Kamileri I. Lugli N. Evangelou K. Da-Re C. Huber F. (2016). Mammalian RAD52 functions in break-induced replication repair of collapsed DNA replication forks. *Mol. Cell* 64 1127–1134. 10.1016/j.molcel.2016.10.038 27984746 PMC5179496

[B219] Sozen B. Amadei G. Cox A. Wang R. Na E. Czukiewska S. (2018). Self-assembly of embryonic and two extra-embryonic stem cell types into gastrulating embryo-like structures. *Nat. Cell Biol.* 20 979–989. 10.1038/s41556-018-0147-7 30038254

[B220] Spies J. Lukas C. Somyajit K. Rask M. B. Lukas J. Neelsen K. J. (2019). 53BP1 nuclear bodies enforce replication timing at under-replicated DNA to limit heritable DNA damage. *Nat. Cell Biol.* 21 487–497. 10.1038/s41556-019-0293-6 30804506

[B221] Stewart G. S. Wang B. Bignell C. R. Taylor A. M. Elledge S. J. (2003). MDC1 is a mediator of the mammalian DNA damage checkpoint. *Nature* 421 961–966. 10.1038/nature01446 12607005

[B222] Stracker T. H. Petrini J. H. J. (2011). The MRE11 complex: starting from the ends. *Nat. Rev. Mol. Cell Biol.* 12 90–103. 10.1038/nrm3047 21252998 PMC3905242

[B223] Strzalka W. Ziemienowicz A. (2011). Proliferating cell nuclear antigen (PCNA): a key factor in DNA replication and cell cycle regulation. *Ann. Bot.* 107 1127–1140. 10.1093/aob/mcq243 21169293 PMC3091797

[B224] Symington L. S. (2016). Mechanism and regulation of DNA end resection in eukaryotes. *Crit. Rev. Biochem. Mol. Biol.* 51 195–212. 10.3109/10409238.2016.1172552 27098756 PMC4957645

[B225] Taglialatela A. Alvarez S. Leuzzi G. Sannino V. Ranjha L. Huang J. W. (2017). Restoration of replication fork stability in BRCA1- and BRCA2-deficient cells by inactivation of SNF2-family fork remodelers. *Mol. Cell* 68 414–430.e8. 10.1016/j.molcel.2017.09.036 29053959 PMC5720682

[B226] Takai H. Tominaga K. Motoyama N. Minamishima Y. A. Nagahama H. Tsukiyama T. (2000). Aberrant cell cycle checkpoint function and early embryonic death in Chk1(-/-) mice. *Genes Dev.* 14 1439–1447. 10859163 PMC316691

[B227] Takeuchi A. Iida K. Tsubota T. Hosokawa M. Denawa M. Brown J. B. (2018). Loss of Sfpq Causes Long-Gene Transcriptopathy in the Brain. *Cell Rep.* 23 1326–1341. 10.1016/j.celrep.2018.03.141 29719248

[B228] Tam P. P. (1989). Regionalisation of the mouse embryonic ectoderm: allocation of prospective ectodermal tissues during gastrulation. *Development* 107 55–67. 2627894 10.1242/dev.107.1.55

[B229] Tam P. P. Williams E. A. Chan W. Y. (1993). Gastrulation in the mouse embryo: ultrastructural and molecular aspects of germ layer morphogenesis. *Microsc. Res. Tech.* 26 301–328. 10.1002/jemt.1070260405 8305722

[B230] Tam P. P. L. Nelson J. Rossant J. (2013). *Mammalian Development.* Cold Spring Harbor, NY: Cold Spring Harbor Laboratory Press.

[B231] Tebbs R. S. Flannery M. L. Meneses J. J. Hartmann A. Tucker J. D. Thompson L. H. (1999). Requirement for the Xrcc1 DNA base excision repair gene during early mouse development. *Dev. Biol.* 208 513–529. 10.1006/dbio.1999.9232 10191063

[B232] Ter Huurne M. Chappell J. Dalton S. Stunnenberg H. G. (2017). Distinct cell-cycle control in two different states of mouse pluripotency. *Cell Stem Cell* 21 449–455.e4. 10.1016/j.stem.2017.09.004 28985526 PMC5658514

[B233] Tesar P. J. Chenoweth J. G. Brook F. A. Davies T. J. Evans E. P. Mack D. L. (2007). New cell lines from mouse epiblast share defining features with human embryonic stem cells. *Nature* 448 196–199. 10.1038/nature05972 17597760

[B234] Thangavel S. Berti M. Levikova M. Pinto C. Gomathinayagam S. Vujanovic M. (2015). DNA2 drives processing and restart of reversed replication forks in human cells. *J. Cell Biol.* 208 545–562. 10.1083/jcb.201406100 25733713 PMC4347643

[B235] Theunissen J. W. Kaplan M. I. Hunt P. A. Williams B. R. Ferguson D. O. Alt F. W. (2003). Checkpoint failure and chromosomal instability without lymphomagenesis in Mre11(ATLD1/ATLD1) mice. *Mol. Cell* 12 1511–1523. 10.1016/s1097-2765(03)00455-6 14690604

[B236] Tichy E. D. Pillai R. Deng L. Liang L. Tischfield J. Schwemberger S. J. (2010). Mouse embryonic stem cells, but not somatic cells, predominantly use homologous recombination to repair double-strand DNA breaks. *Stem Cells Dev.* 19 1699–1711. 10.1089/scd.2010.0058 20446816 PMC3128311

[B237] Toledo L. I. Altmeyer M. Rask M. B. Lukas C. Larsen D. H. Povlsen L. K. (2013). ATR prohibits replication catastrophe by preventing global exhaustion of RPA. *Cell* 155 1088–1103. 10.1016/j.cell.2013.10.043 24267891

[B238] Tominaga Y. Li C. Wang R.-H. Deng C.-X. (2006). Murine Wee1 plays a critical role in cell cycle regulation and pre-implantation stages of embryonic development. *Int. J. Biol. Sci.* 2 161–170. 10.7150/ijbs.2.161 16810330 PMC1483124

[B239] Tomoda K. Yoneda-Kato N. Fukumoto A. Yamanaka S. Kato J. Y. (2004). Multiple functions of Jab1 are required for early embryonic development and growth potential in mice. *J. Biol. Chem.* 279 43013–43018. 10.1074/jbc.M406559200 15299027

[B240] Tsuzuki T. Fujii Y. Sakumi K. Tominaga Y. Nakao K. Sekiguchi M. (1996). Targeted disruption of the Rad51 gene leads to lethality in embryonic mice. *Proc. Natl. Acad. Sci. U.S.A.* 93 6236–6240. 10.1073/pnas.93.13.6236 8692798 PMC39005

[B241] Uchimura A. Hidaka Y. Hirabayashi T. Hirabayashi M. Yagi T. (2009). DNA polymerase delta is required for early mammalian embryogenesis. *PLoS One* 4:e4184. 10.1371/journal.pone.0004184 19145245 PMC2615215

[B242] Uziel T. Lerenthal Y. Moyal L. Andegeko Y. Mittelman L. Shiloh Y. (2003). Requirement of the MRN complex for ATM activation by DNA damage. *EMBO J.* 22 5612–5621. 10.1093/emboj/cdg541 14532133 PMC213795

[B243] Vallabhaneni H. Lynch P. J. Chen G. Park K. Liu Y. Goehe R. (2018). High basal levels of gammaH2AX in human induced pluripotent stem cells are linked to replication-associated DNA damage and repair. *Stem Cells* 36 1501–1513. 10.1002/stem.2861 29873142 PMC6662168

[B244] Valnegri P. Huang J. Yamada T. Yang Y. Mejia L. A. Cho H. Y. (2017). RNF8/UBC13 ubiquitin signaling suppresses synapse formation in the mammalian brain. *Nat. Commun.* 8:1271. 10.1038/s41467-017-01333-6 29097665 PMC5668370

[B245] van Harten A. M. Buijze M. Van Der Mast R. Rooimans M. A. Martens-De Kemp S. R. Bachas C. (2019). Targeting the cell cycle in head and neck cancer by Chk1 inhibition: a novel concept of bimodal cell death. *Oncogenesis* 8:38. 10.1038/s41389-019-0147-x 31209198 PMC6572811

[B246] Van Ly D. Low R. R. J. Frolich S. Bartolec T. K. Kafer G. R. Pickett H. A. (2018). Telomere loop dynamics in chromosome end protection. *Mol. Cell* 71 510–525.e6. 10.1016/j.molcel.2018.06.025 30033372

[B247] Wachowicz P. Fernández-Miranda G. Marugán C. Escobar B. De Cárcer G. (2016). Genetic depletion of Polo-like kinase 1 leads to embryonic lethality due to mitotic aberrancies. *Bioessays* 38 (Suppl. 1), S96–S106. 10.1002/bies.201670908 27417127

[B248] Wallace S. S. (2014). Base excision repair: a critical player in many games. *DNA Repair* 19 14–26. 10.1016/j.dnarep.2014.03.030 24780558 PMC4100245

[B249] Wang X. Chang Y. Li Y. Zhang X. Goodrich D. W. (2006). Thoc1/Hpr1/p84 is essential for early embryonic development in the mouse. *Mol. Cell. Biol.* 26 4362–4367. 10.1128/MCB.02163-05 16705185 PMC1489088

[B250] Wang Y. Putnam C. D. Kane M. F. Zhang W. Edelmann L. Russell R. (2005). Mutation in Rpa1 results in defective DNA double-strand break repair, chromosomal instability and cancer in mice. *Nat. Genet.* 37 750–755. 10.1038/ng1587 15965476

[B251] Wang Z. Q. Auer B. Stingl L. Berghammer H. Haidacher D. Schweiger M. (1995). Mice lacking ADPRT and poly(ADP-ribosyl)ation develop normally but are susceptible to skin disease. *Genes Dev.* 9 509–520. 10.1101/gad.9.5.509 7698643

[B252] Ward I. M. Minn K. Van Deursen J. Chen J. (2003). p53 Binding protein 53BP1 is required for DNA damage responses and tumor suppression in mice. *Mol. Cell. Biol.* 23 2556–2563. 10.1128/mcb.23.7.2556-2563.2003 12640136 PMC150747

[B253] Watanabe N. Arai H. Nishihara Y. Taniguchi M. Watanabe N. Hunter T. (2004). M-phase kinases induce phospho-dependent ubiquitination of somatic Wee1 by SCFbeta-TrCP. *Proc. Natl. Acad. Sci. U.S.A.* 101 4419–4424. 10.1073/pnas.0307700101 15070733 PMC384762

[B254] Watson C. M. Tam P. P. (2001). Cell lineage determination in the mouse. *Cell Struct. Funct.* 26 123–129. 11565804 10.1247/csf.26.123

[B255] Weiss R. S. Enoch T. Leder P. (2000). Inactivation of mouse Hus1 results in genomic instability and impaired responses to genotoxic stress. *Genes Dev.* 14 1886–1898. 10921903 PMC316817

[B256] Wen B. Li R. Cheng K. Li E. Zhang S. Xiang J. (2017). Tetraploid embryonic stem cells can contribute to the development of chimeric fetuses and chimeric extraembryonic tissues. *Sci. Rep.* 7:3030. 10.1038/s41598-017-02783-0 28596585 PMC5465063

[B257] Wilhelm T. Olziersky A.-M. Harry D. De Sousa F. Vassal H. Eskat A. (2019). Mild replication stress causes chromosome mis-segregation via premature centriole disengagement. *Nat. Commun.* 10:3585. 10.1038/s41467-019-11584-0 31395887 PMC6687892

[B258] Xanthoudakis S. Smeyne R. J. Wallace J. D. Curran T. (1996). The redox/DNA repair protein, Ref-1, is essential for early embryonic development in mice. *Proc. Natl. Acad. Sci. U.S.A.* 93 8919–8923. 10.1073/pnas.93.17.8919 8799128 PMC38569

[B259] Xiao Y. Weaver D. T. (1997). Conditional gene targeted deletion by Cre recombinase demonstrates the requirement for the double-strand break repair Mre11 protein in murine embryonic stem cells. *Nucleic Acids Res.* 25 2985–2991. 10.1093/nar/25.15.2985 9224597 PMC146850

[B260] Xiao Z. Chen Z. Gunasekera A. H. Sowin T. J. Rosenberg S. H. Fesik S. (2003). Chk1 mediates S and G2 arrests through Cdc25A degradation in response to DNA-damaging agents. *J. Biol. Chem.* 278 21767–21773. 10.1074/jbc.M300229200 12676925

[B261] Xie R. Medina R. Zhang Y. Hussain S. Colby J. Ghule P. (2009). The histone gene activator HINFP is a nonredundant cyclin E/CDK2 effector during early embryonic cell cycles. *Proc. Natl. Acad. Sci. U.S.A.* 106 12359–12364. 10.1073/pnas.0905651106 19590016 PMC2718389

[B262] Xu Y. Ashley T. Brainerd E. E. Bronson R. T. Meyn M. S. Baltimore D. (1996). Targeted disruption of ATM leads to growth retardation, chromosomal fragmentation during meiosis, immune defects, and thymic lymphoma. *Genes Dev.* 10 2411–2422. 10.1101/gad.10.19.2411 8843194

[B263] Yamazaki S. Ishii A. Kanoh Y. Oda M. Nishito Y. Masai H. (2012). Rif1 regulates the replication timing domains on the human genome. *EMBO J.* 31 3667–3677. 10.1038/emboj.2012.180 22850674 PMC3442267

[B264] Yang W. Klaman L. D. Chen B. Araki T. Harada H. Thomas S. M. (2006). An Shp2/SFK/Ras/Erk signaling pathway controls trophoblast stem cell survival. *Dev. Cell* 10 317–327. 10.1016/j.devcel.2006.01.002 16516835

[B265] Yata K. Bleuyard J. Y. Nakato R. Ralf C. Katou Y. Schwab R. A. (2014). BRCA2 coordinates the activities of cell-cycle kinases to promote genome stability. *Cell Rep.* 7 1547–1559. 10.1016/j.celrep.2014.04.023 24835992 PMC4062933

[B266] Yonemasu R. Minami M. Nakatsu Y. Takeuchi M. Kuraoka I. Matsuda Y. (2005). Disruption of mouse XAB2 gene involved in pre-mRNA splicing, transcription and transcription-coupled DNA repair results in preimplantation lethality. *DNA Repair* 4 479–491. 10.1016/j.dnarep.2004.12.004 15725628

[B267] Yoon J. C. Ling A. J. Isik M. Lee D. Y. Steinbaugh M. J. Sack L. M. (2014). GLTSCR2/PICT1 links mitochondrial stress and Myc signaling. *Proc. Natl. Acad. Sci. U.S.A.* 111 3781–3786. 10.1073/pnas.1400705111 24556985 PMC3956149

[B268] Yoshida K. Kuo F. George E. L. Sharpe A. H. Dutta A. (2001). Requirement of CDC45 for postimplantation mouse development. *Mol. Cell. Biol.* 21 4598–4603. 10.1128/MCB.21.14.4598-4603.2001 11416137 PMC87121

[B269] Zaugg K. Su Y. W. Reilly P. T. Moolani Y. Cheung C. C. Hakem R. (2007). Cross-talk between Chk1 and Chk2 in double-mutant thymocytes. *Proc. Natl. Acad. Sci. U.S.A.* 104 3805–3810. 10.1073/pnas.0611584104 17360434 PMC1820665

[B270] Zaveri L. Dhawan J. (2018). Cycling to meet fate: connecting pluripotency to the cell cycle. *Front. Cell Dev. Biol.* 6:57. 10.3389/fcell.2018.00057 29974052 PMC6020794

[B271] Zellweger R. Dalcher D. Mutreja K. Berti M. Schmid J. A. Herrador R. (2015). Rad51-mediated replication fork reversal is a global response to genotoxic treatments in human cells. *J. Cell Biol.* 208 563–579. 10.1083/jcb.201406099 25733714 PMC4347635

[B272] Zeman M. K. Cimprich K. A. (2014). Causes and consequences of replication stress. *Nat. Cell Biol.* 16 2–9. 10.1038/ncb2897 24366029 PMC4354890

[B273] Zhang Y. Hunter T. (2014). Roles of Chk1 in cell biology and cancer therapy. *Int. J. Cancer* 134 1013–1023. 10.1002/ijc.28226 23613359 PMC3852170

[B274] Zhang Y. Zolov S. N. Chow C. Y. Slutsky S. G. Richardson S. C. Piper R. C. (2007). Loss of Vac14, a regulator of the signaling lipid phosphatidylinositol 3,5-bisphosphate, results in neurodegeneration in mice. *Proc. Natl. Acad. Sci. U.S.A.* 104 17518–17523. 10.1073/pnas.0702275104 17956977 PMC2077288

[B275] Zhao B. Zhang W. Cun Y. Li J. Liu Y. Gao J. (2018). Mouse embryonic stem cells have increased capacity for replication fork restart driven by the specific Filia-Floped protein complex. *Cell Res.* 28 69–89. 10.1038/cr.2017.139 29125140 PMC5752841

[B276] Zhao B. Zhang W. D. Duan Y. L. Lu Y. Q. Cun Y. X. Li C. H. (2015). Filia Is an ESC-Specific Regulator of DNA Damage Response and Safeguards Genomic Stability. *Cell Stem Cell* 16 684–698. 10.1016/j.stem.2015.03.017 25936915 PMC4610728

[B277] Zheng P. (2020). Maintaining genomic stability in pluripotent stem cells. *Genome Instabil. Dis.* 1 92–97.

[B278] Zhou Z. Wang L. Ge F. Gong P. Wang H. Wang F. (2018). Pold3 is required for genomic stability and telomere integrity in embryonic stem cells and meiosis. *Nucleic Acids Res.* 46 3468–3486. 10.1093/nar/gky098 29447390 PMC6283425

[B279] Zhu J. Petersen S. Tessarollo L. Nussenzweig A. (2001). Targeted disruption of the Nijmegen breakage syndrome gene NBS1 leads to early embryonic lethality in mice. *Curr. Biol.* 11 105–109. 10.1016/s0960-9822(01)00019-7 11231126

[B280] Ziegler-Birling C. Helmrich A. Tora L. Torres-Padilla M.-E. (2009). Distribution of p53 binding protein 1 (53BP1) and phosphorylated H2A.X during mouse preimplantation development in the absence of DNA damage. *Int. J. Dev. Biol.* 53 1003–1011. 10.1387/ijdb.082707cz 19598117

[B281] Zimmermann M. Lottersberger F. Buonomo S. B. Sfeir A. De Lange T. (2013). 53BP1 regulates DSB repair using Rif1 to control 5′ end resection. *Science* 339 700–704. 10.1126/science.1231573 23306437 PMC3664841

[B282] Zong D. Adam S. Wang Y. Sasanuma H. Callen E. Murga M. (2019). BRCA1 haploinsufficiency is masked by RNF168-mediated chromatin ubiquitylation. *Mol. Cell* 73 1267–1281.e7. 10.1016/j.molcel.2018.12.010 30704900 PMC6430682

